# Pharmacological inhibition of P2RX7 ameliorates liver injury by reducing inflammation and fibrosis

**DOI:** 10.1371/journal.pone.0234038

**Published:** 2020-06-03

**Authors:** Bernat Baeza-Raja, Andrew Goodyear, Xiao Liu, Kevin Lam, Lynn Yamamoto, Yingwu Li, G. Steven Dodson, Toshi Takeuchi, Tatiana Kisseleva, David A. Brenner, Karim Dabbagh

**Affiliations:** 1 Second Genome Inc., South San Francisco, California, United States of America; 2 Department of Surgery, University of California San Diego, La Jolla, California, United States of America; 3 Department of Medicine, University of California San Diego, La Jolla, California, United States of America; University of Navarra School of Medicine and Center for Applied Medical Research (CIMA), SPAIN

## Abstract

Extracellular adenosine triphosphate (eATP) released by damaged cells, and its purinergic receptors, comprise a crucial signaling network after injury. Purinergic receptor P2X7 (P2RX7), a major driver of NOD-like receptor family pyrin domain containing 3 (NLRP3) inflammasome activation and IL-1β processing, has been shown to play a role in liver injury in murine diet- and chemically-induced liver injury models. It is unclear, however, whether P2RX7 plays a role in non-alcoholic steatohepatitis (NASH) and which cell type is the main target of P2RX7 pharmacological inhibition. Here, we report that P2RX7 is expressed by infiltrating monocytes and resident Kupffer cells in livers from NASH-affected individuals. Using primary isolated human cells, we demonstrate that P2RX7 expression in CD14^+^ monocytes and Kupffer cells primarily mediates IL-1β release. In addition, we show that pharmacological inhibition of P2RX7 in monocytes and Kupffer cells, blocks IL-1β release, reducing hepatocyte caspase 3/7 activity, IL-1β-mediated CCL2 and CCL5 chemokine gene expression and secretion, and hepatic stellate cell (HSC) procollagen secretion. Consequently, in a chemically-induced nonhuman primate model of liver fibrosis, treatment with a P2RX7 inhibitor improved histological characteristics of NASH, protecting from liver inflammation and fibrosis. Taken together, these findings underscore the critical role of P2RX7 in the pathogenesis of NASH and implicate P2RX7 as a promising therapeutic target for the management of this disease.

## Introduction

Hepatocyte injury, inflammation, and immune cell activation are common features of the pathogenesis of most liver diseases, including non-alcoholic steatohepatitis (NASH) [1]. Persistent and progressive hepatic inflammation has been associated with the development of liver fibrosis [2,3], which can lead to cirrhosis, hepatocellular carcinoma, and liver failure [4]. As such, controlling the early onset of hepatic inflammation might preclude the development of severe liver diseases. As NASH is predicted to be the leading cause of liver transplantation over the next decade and approved pharmacological therapies remain unavailable [5,6], it is of critical and timely importance to identify therapeutic targets to effectively treat this condition.

Liver inflammation is known to be triggered by pathogens and danger signals derived from the host. Changes in the intestinal mucosal permeability and increased bacterial translocation result in increased plasma and portal circulatory levels of molecules derived from those pathogens, known as pathogen-associated molecular patterns (PAMPs) such as lipopolysaccharide (LPS), which initiate a transcriptional inflammatory response through the activation of Toll-like receptors (TLR) on immune cells in the liver. In addition, a second signal provided by the so-called damage-associated molecular patterns (DAMPs) such as extracellular adenosine triphosphate (eATP), released from damaged parenchymal and nonparenchymal cells, leads to the assembly of an intracellular protein complex termed ‘the Inflammasome’. The combination of these signals trigger caspase-1 activation and the generation of proinflammatory cytokines IL-1β and IL-18 [7,8] which contribute to the amplification of inflammation by activating resident macrophages (i.e. Kupffer cells) and recruiting bone marrow-derived monocytes and macrophages to the site of injury. In turn, infiltrating monocyte/macrophages amplify this immune response by producing inflammatory cytokines and chemokines which further promote recruitment of inflammatory cells [9]. Simultaneously, hepatocyte death and the inflammatory response initiate the development of fibrosis by upregulating the activation of hepatic stellate cells (HSCs) into myofibroblasts, which are the primary source of scar-forming matrix proteins [10–13]. Emerging data have provided evidence for the role of NOD-like receptor family pyrin domain containing 3 (NLRP3) inflammasome [14–16], caspase-1[17,18] and IL-1β [19–23] as major contributors to hepatocyte damage, immune cell activation and amplification of liver inflammation and fibrosis in several liver injury murine models. However, little is known about the role of the purinergic receptor P2X7 (P2RX7), a major driver of NLRP3 inflammasome activation and IL-1β processing [24], in liver inflammation and fibrosis. The purinergic receptor P2X7 (P2RX7), a member of the ligand ATP-gated ionotropic P2X class of receptors, is activated by high concentrations of extracellular ATP, released by damaged or stressed cells during injury, constituting a powerful signal which enhances liver inflammation and fibrosis [24]. This activation triggers cytoplasmic ion transport (Na^+^ and Ca^+^ influx, K^+^ efflux) [25], which induces the NLRP3 inflammasome complex activation and subsequent release of mature IL-1β [26,27]. While genetic ablation of *P2rx7* has been shown to reduce metabolic oxidative stress and autophagy in murine liver injury models [28,29], the role of P2RX7 in NASH and its contribution to liver cells functions remains elusive.

To this end, we aimed to identify the role and source of P2RX7 in NASH, investigate the effects of P2RX7 inhibition on human liver cells and evaluate its potential as a therapeutic target for liver injury in non-human primates. Here, we report that NASH-affected livers have a greater number of cells expressing P2RX7, and this increase was predominantly associated with infiltrating monocytes (MOs) and resident Kupffer cells (KCs) in the livers of NASH-affected individuals. Pharmacological inhibition of P2RX7 in human primary CD14^+^ MOs and KCs primarily blocked IL-1β release, but also modulated the secretion of several proinflammatory cytokines triggered by P2RX7 in MOs. Reduced P2RX7-dependent IL-1β secretion from MOs and KCs resulted in decreased hepatocyte damage, chemokine secretion, and HSCs fibrosis. The relevance of the *in vitro* findings was evaluated *in vivo* via pharmacological inhibition of P2RX7 with SGM-1019. P2RX7 inhibition resulted in significant protection from inflammation and fibrosis in a chemically-induced primate (non-human) liver fibrosis model. Collectively, the findings of this study demonstrate the great potential of pharmacological inhibition of P2RX7 as a novel therapy for NASH.

## Materials and methods

### Human liver samples

Slides consisting of two sections of liver tissue per slide, from a variety of NASH donor tissues were obtained from Samsara Sciences, Inc., San Diego, CA. Samsara Sciences provided slides from whole livers that were obtained through IIAM from deceased donors who had been consented for transplantation and research use according to all federal, state and institutional regulations. Only whole livers that were deemed non-transplantable are processed to isolate the individual liver cell types, snap-frozen tissue and FFPE-blocks. All livers were tested and found negative for HIV I/II, HBV, HCV and syphilis. Donors with excessive alcohol consumption (>140 g for men or >70g for women, per week), and/or *i*.*v*. drug use were excluded from the study. Liver samples from suspected NASH and control donors were collected from excess tissue not used in the cell isolation process. Sections were cut from a representative paraffin block, mounted on glass slides and stained with hematoxylin/eosin (H&E) or trichrome (TC). Histopathologic assessment was conducted on the H&E and TC stained slides by a board-certified pathologist and scored according to standard clinical practices. Inflammation and fibrosis were assessed using standard Batts-Ludwig scoring methodology (Scale 0–4) and a NAFLD score (NAS) was assigned according to the standards of the NASH CRN Scoring System [30]. Clinical and histological characteristics of these samples are provided in **S1 Table** in the supplementary information.

### Isolation of ALD and normal human HSCs

Livers declined for transplantation (IRB 171883XX approved on 11/9/17, obtained via collaboration with OPO Lifesharing, www.lifesharing.org), were graded for steatosis, inflammation, and fibrosis by a pathologist using a double-blinded method, and identified as ALD or normal. A liver pathologist assessed the histology in a double blinded manner. Primary human HSCs were purified from livers using pronase/collagenase perfusion and gradient centrifugation method,[31] cultured for 3 weeks (P0), passaged once (P1) or twice (P2), and analyzed by qPCR.

### Cell lines and treatment

Human primary hepatocytes, HSCs, and KCs were purchased from Samsara Sciences, Inc. Hepatocytes were thawed with hepatocyte thaw media (#CM7500, Life Technologies), plated in William’s medium E (#A12176-01, Life Technologies) with 1% L-glutamine (#35050–061, Life Technologies), 1% of Antibiotic/Antimycotic solution (#400–101, Gemini Biologicals), and 5% of fetal bovine serum (FBS) (#35-016-CV, Corning), and maintained in William’s medium E (#A12176-01, Life Technologies) containing 1% of insulin-transferrin-selenium (#41400–045, Life Technologies), 0.1 μM of dexamethasone (#D4902, Sigma-Aldrich), 1% of Antibiotic/Antimycotic solution (#400–101, Gemini Biologicals) and 10 mM Hepes (25-060-CI, Corning). HSCs were maintained for 24 hours in DMEM/Ham’s F12 with Hepes (#11330, ThermoFisher Scientific) containing 10% of FBS (#35-016-CV, Corning) and penicillin/streptomycin before depleting serum from media for 24 hours before treatments. KCs were maintained in RPMI 1640, Glutamax with Hepes (72400, ThermoFisher Scientific) with 10% of FBS (#35-016-CV, Corning) and penicillin/streptomycin. Hepatocytes, HSCs, and KCs were seeded on collagen-coated plates. Human primary CD14^+^ MOs were isolated from peripheral blood mononuclear cells (PBMCs) from fresh blood donations from the Stanford Blood Center. PBMCs were isolated from healthy individuals using density gradient centrifugation with Ficoll-Paque PLUS (GE Healthcare) according to the manufacturer’s instructions. CD14^+^ MOs were isolated with the human CD14^+^ cell isolation kit (#130-050-201, Miltenyi Biotech). Cell numbers were assessed by Neubauer chamber counting.

To examine inflammasome activation, KCs and CD14^+^ MOs were cultured in the presence of 0.3–0.9 and 3 mM of ATP, respectively for 45´ after LPS was administered at 100 ng/ml for 18 and 1 hour, respectively. For P2RX7 inhibition, 1 μM of SGM-1019 was added 15´ before the addition of ATP. Human primary HSCs and hepatocytes were cultured with recombinant human IL-1β (#200-01B, Prepotech) for 18 hours in the presence or absence of LPS. Staurosporine was used at 1 μM. Human primary HSCs from control and NASH-affected donor were treated with 10 ng/ml of recombinant human IL-1β. Conditioned media (CM) from human primary KCs (CM-KCs) or CD14^+^ MOs (CM-MOs) treated with LPS and ATP with or without SGM-1019 at the indicated times were used to culture human primary hepatocytes and HSCs for 24 hours. CM was collected, centrifuged and stored at -80°C until its use on the indicated cells. Human primary HSCs and hepatocytes were cultured with 100 ng/ml of recombinant human IL-1Ra (#SRP3327, Sigma) for 1 hour before culture with CM. IL-1Ra was also added in the CM until collected at the same concentration.

### qPCR analysis

For qPCR assays, total RNA was isolated using the PureLink^®^ RNA Kit (#12183025, Ambion) according to the manufacturer’s instructions. RNA was reverse transcribed to cDNA using the Maxima First Strand cDNA Synthesis Kit (#K1671, ThermoFisher Scientific) according to the manufacturer’s instructions. Taqman (#4444554, Applied Biosystems) was used to quantify the PCR-amplification products. The mRNA expression levels of the target genes were normalized to *HPRT1* expression. The primers used in this study were obtained from ThermoFisher Scientific: *P2RX7* (Hs00175721_m1), *NLRP3* (Hs00918082_m1), *AIM2* (Hs00915710_m1), ASC1 (Hs1547324_gH), *CASP1* (Hs00354836_m1), *IL-1β* (Hs01555410_m1), *COL1α1* (Hs00164004_m1), *COL1α2* (Hs01028956_m1), *COL4α1* (Hs00266237_m1), *ACTA2* (Hs00426835_g1), *TGFβ1* (Hs00998133_m1), *CD11b* (Hs00167304_m1), *CD45* (Hs02519237_s1), *TNFα* (Hs00174128_m1), *CCL2* (Hs00234140_m1), *CCL5* (Hs00982282_m1), *CD14* (Hs02621496_s1), *CD68* (Hs02836816_g1), *DR5* (Hs00366278_m1), *DDIT3* (Hs00358796_g1), *ERO1α* (Hs00205880_m1), *GADD34* (Hs00169585_m1), *ENTPD1* (Hs00969556_m1), *NT5E* (Hs00159686_m1), and *HPRT1* (#4325801).

### Immunohistochemical staining

Paraffin-embedded tissue sections were deparaffinized and rehydrated in an ethanol series to water, followed by heat-induced epitope retrieval for 20 min using a pressure cooker (Biocare medical) in antigen retrieval solution (1:10, #CB910M, Biocare medical). Sections were blocked for endogenous peroxidase with 3% H_2_O_2_ for 10’ and further were blocked for non-specific binding with protein block serum-free (#X0909, Agilent,) for 15′. Slides were then incubated at room temperature for 1 hour with the following antibodies: anti-P2RX7 antibody (1:500, PA5-19165, ThermoFisher Scientific); anti-CD45 / LCA antibody (1:300, #LS-C340234 Clone UCH, LSBio); anti-CD 68 antibody (KP1) (1:100, #ab955, Abcam). They were visualized using secondary antibody mouse-on-mouse or/and rabbit-on-rodent (Biocare Medical) with diaminobenzidine (DAB) for HRP polymer or wrap red for alkaline phosphatase (AP) polymer. Anti-CD14 biotinylated antibody (1:40, #BAF383, R&D system) was visualized using streptavidin alkaline phosphatase (1:250, #SA-5100, Vector Laboratory) with wrap red. All the slides were counterstained with hematoxylin. Sections processed with replacement of the primary antibody by protein block were used as a negative control. Tris-buffered saline with 0.1% Tween 20 was used as a wash buffer. Normal human tonsil tissue was used as a positive control. Microscopic images were acquired with Keyence microscope BZ-X700 (Keyence Corporation). Percentage of stained-positive cells was obtained by using ImageJ software with cell counter plugin by quantifying 3–8 images per section (2–4 sections per liver). We determined the number of stained-positive cells by counting colored stained-positive cells and total cell numbers.

### Histological analysis

Liver sections were embedded in paraffin and then stained using hematoxylin and eosin (H&E) to visualize the pattern of lipid accumulation and the inflammatory status of the tissues. Sirius red staining was performed to evaluate tissue fibrosis. H&E and Sirius red staining were performed according to standard protocols. Microscopic images were acquired with Keyence microscope BZ-X700 (Keyence Corporation). Percentage of Sirius red-positive area was obtained by using ImageJ software by quantifying 5–6 images per section (2–6 sections per liver).

#### Luminex–eBioscience/Affymetrix magnetic bead kits

Levels of cytokines in the supernatants from human primary CD14^+^ monocytes and Kupffer cells were measured using a 63-plex Luminex antibody-conjugated bead capture assay (Affymetrix). This assay was performed in the Human Immune Monitoring Center at Stanford University. Human 63-plex kits were purchased from eBiosciences/Affymetrix and used according to the manufacturer’s recommendations with modifications as described below. Briefly, beads were added to a 96 well plate and washed in a Biotek ELx405 washer. Samples were added to the plate containing the mixed antibody-linked beads and incubated at room temperature for 1 hour followed by overnight incubation at 4°C with shaking. Cold and Room temperature incubation steps were performed on an orbital shaker at 500–600 rpm. Following the overnight incubation plates were washed in a Biotek ELx405 washer and then biotinylated detection antibody added for 75 minutes at room temperature with shaking. Plate was washed as above and streptavidin-PE was added. After incubation for 30 minutes at room temperature wash was performed as above and reading buffer was added to the wells. Each sample was measured in duplicate. Plates were read using a Luminex 200 instrument with a lower bound of 50 beads per sample per cytokine. Custom assay Control beads by Radix Biosolutions are added to all wells.

### ELISA assays

IL-1β, CCL2, CCL5, procollagen, caspase-1, and ENTPD1 levels were measured using the Human IL-1β/IL-1F2 Immunoassay Quantikine^®^ ELISA (#DLB50, R&D Systems), human CCL2/MCP-1 Quantikine^®^ ELISA kit (#DCP100, R&D Systems), human CCL5/RANTES Quantikine^®^ ELISA kit (#DRN00B, R&D Systems), human Pro-Collagen Iα1 DuoSet ELISA (#DY6220-05, R&D Systems), human Caspase-1 ELISA kit (ab219633, Abcam), and human CD39/ENTPD1 ELISA (#DY4397, R&D Systems) following manufacturer’s instructions. Caspase 3/7 activity and cell viability were measured by using the Caspase-Glo^®^ 3/7 Assay (#G8091, Promega) and MTT Assay kit (ab211091, Abcam) following manufacturer’s instructions.

### Whole blood assays

Blood from healthy human volunteers was obtained from Stanford Blood Center. All blood donations were approved by the ethical committee at Stanford University (IRB 7 #5136). All blood donors were provided documentation informing them to potential risks of blood donation, conditions precluding donation and possible side effects. Written consent approving use of blood for research purposes including investigational research tests was obtained from all donors. All donors under the age of 17 required signed consent of a parent or guardian, as approved the ethics committee. Blood from cynomolgus macaques (Valley Biosystems) and human blood was collected in 10 mL vacutainer tubes spray coated with sodium heparin (BD Biosciences), maintained at room temperature and used within 2–3 hours of collection. A total volume of 200 μl undiluted whole blood was used. Twenty-five μl of SGM-1019 dissolved in DMSO (Corning) and diluted in RPMI (ThermoFisher Scientific) were added to obtain a final concentration of 3.0, 1.5, 0.75, 0.38, 0.19 0.094 or 0 μM. Concurrent with the addition of SGM-1019, LPS (Invivogen) was added to each well obtain a final concentration of 100 ng/ml. Blood was incubated at 37°C for 1 hour (human) or 2 hours (primates). ATP (VWR) diluted in 25 mM HEPES pH 7.0 (Thermo Fisher Scientific) was added to obtain a final concentration of 20, 10, 5, 2.5, 1.25, 0.63, 0.3 or 0 mM. Blood was incubated at 37°C for 45 minutes. Plasma was collected by centrifuging at 1000g for 2 minutes at room temperature and frozen at -80°C until analyzed. Plasma samples were pre-diluted in 4% BSA (w/v) (Millipore-Sigma) in PBS (Corning) as needed before analysis of IL-1β by ELISA. IL-1β pg/mL concentrations observed in the presence of various SGM-1019 concentrations were normalized to maximal IL-1β pg/mL responses measured in vehicle (DMSO) treated blood (E/E_max_). AUC analysis was then performed on each agonist response curve and a regression analysis of AUC values was performed to determine SGM-1019 concentrations resulting in 50 and 95% inhibition (IC_50_ and IC_95_) of IL-1β secretion.

### Non-human primate study

Study was performed at HD Biosciences Co., Ltd. Forty-eight male cynomolgus primates at the age of 4–6 years old and 4–6 kg of body weight were purchased from Guangdong Landao LTD. Animals were housed in individual cages with sufficient space for 1 week of acclimation prior to the experiment in individual cages measuring 80 cm (length) × 80 cm (width) × 80 cm (height) stainless steel cage with perch and horizontal opening for socialization was used to house one monkey for acclimation. Then, monkeys were housed in groups of 4 monkeys for 2 weeks prior to the experiment in 200 cm (length) × 200 cm (width) × 220 cm (height) stainless steel cages with perch, swing and bracket were used to house four monkeys. As much as possible, monkeys will be housed in groups or pairs and cages are arranged in such a way that the animals have visual contact with each other. Cages were maintained in a standard environment of 20-25°C of room temperature, 40–70% relative humidity and natural lighting. Water was available *ad libitum* and standard food was supplemented with vegetables and fruits (fresh and dry). In addition, monkeys were provided with novel toys and manipulative devices that encourage species-typical behavior such as food enrichment (fresh and dried fruits, vegetables and seeds), devices and implement other ways of providing foods to the animals which utilize their cognitive and foraging skills (peanuts, sunflower seeds and currant in balls or pipes with holes or foraging boards), and expand enrichment to the other senses by increasing auditory, visual, and tactile with maneuverable enrichment devices). Fresh weekly prepared CCl_4_ solution (0.5 ml/Kg of CCl_4_ (SinoPharm Chemical, Cat#0000497814) in olive oil (Betis, Cat#0000497814)) was administered by *i*.*p*. injection twice per week (10:00 am Monday and Thursday) during 6 weeks. After 2 weeks, SGM-1019 in 1% hydroxypropylmethyl cellulose (Sigma-Aldrich) solution (provided by Second Genome) and obeticholic acid (OCA, Cat#DC7430, D&C Chemicals) were administered orally BID and QD, respectively for the final 4 weeks of the study. Dosing volumes were 10 ml/kg. Monkeys were carefully monitored and recorded daily by the veterinarian. General condition (evidence of disease, changes in attitude, activity, appetite or behavior suggestive of illness) and detailed and closer inspection of the animal was conducted by gently drawing the animal towards the front of the cage were performed daily and individual body weights were recorded once per week. Conditions for euthanasia can be found in the Supplementary information. At the end of the study, animals were euthanized with Pelltobarbitalum Natricum (Guzhousilekeji LTD) overdose (150 mg/kg *i*.*v*.) and all efforts were made to minimize animal suffering. All samples were collected at the end of the study. All the experimental procedures were approved and performed in strict accordance under the guidelines of the Institutional Animal Care and Use Committee (IACUC) at HD Biosciences Co., Ltd approved by the Shanghai Science and Technology Commission.

### Statistical analysis

All data were analyzed using the appropriate statistical analysis methods, as specified in the figure legends, with the GraphPad Prism software (version 8). Unpaired two-sided Student’s t-tests by non-parametric two-sided Mann-Whitney test were performed to evaluate significance between two experimental groups. One-way ANOVA followed by Tukey’s multiple-comparisons test were done for multiple comparisons. All data are expressed as mean ± SEM. *P* < 0.05 was considered significant. The exact number of animals used in each experiment is presented in the figure legends.

## Results

### P2RX7 is expressed in inflammatory cells during NASH

**Fig 1:**
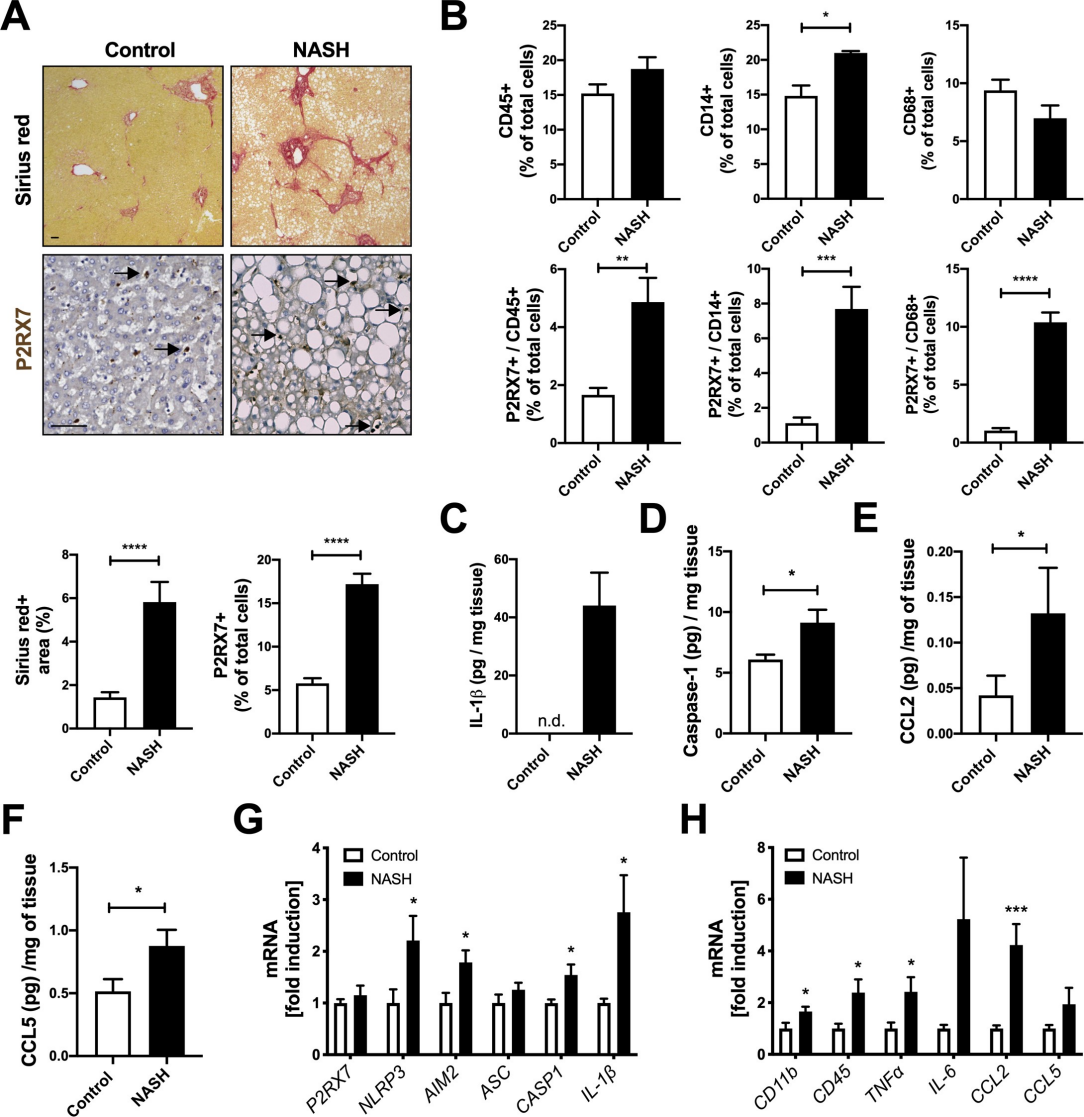
P2RX7 and inflammasome activation are increased in NASH-affected liver. (A) Representative images (objective 2X) of Sirius red staining (top) and immunohistochemical staining (objective 40X) of P2RX7 (bottom) in liver tissue from a representative control and NASH-affected donor with quantification of Sirius red^+^ area and P2RX7^+^ cells (*n* = 5 individuals per group; *n* = 6–8 images per donor). Scale bar, 100 μM. Black arrows highlight P2RX7^+^ cells. (B) Quantification of CD45^+^, CD45^+^/ P2RX7^+^, CD14^+^, CD14^+^/ P2RX7^+^, CD68^+^, CD68^+^/ P2RX7^+^ cells (*n* = 5 individuals per group; *n* = 6–8 images per donor). (C) IL-1β, (D) caspase-1, (E) CCL2, and (F) CCL5 levels in liver tissue from control and NASH-affected liver biopsies (*n* = 5 individuals per group). (G) Relative expression levels of *P2RX*7, *NLRP3*, *AIM2*, *ASC*, *CASP1*, *IL-1β* and (H) *CD11b*, *CD45*, *Tnfα*, *Il-6*, *CCL2*, *CCL5* in liver tissue from control and NASH-affected donors (*n* = 5 individuals per group). n.d., for not detected. In all statistical plots, the data are shown as the mean ± SEM. *P ≤ 0.05, **P ≤ 0.01, ***P ≤ 0.001, ****P ≤ 0.0001 by two-sided Student’s t-test.

To determine whether P2RX7 plays a role in NASH, we compared receptor abundance in liver biopsies from NASH patients and healthy control. As expected, the results of Sirius red staining for collagen presence showed that NASH-affected liver biopsies endured significantly more fibrosis compared to healthy controls (Fig 1A). In addition, a significant increase in the number of P2RX7^+^ cells were observed in the NASH-affected liver biopsies compared to healthy control (Fig 1A), despite similar levels of expressed *P2RX7* mRNA (Fig 1G), suggesting that the observed increase in P2RX7 expression was not due to changes in transcription, but could be attributed to a greater number of cells expressing P2RX7. In fact, P2RX7 is highly expressed in inflammatory and immune cell populations [24], and recruitment of bone marrow-derived monocytes, together with KC activation, plays a major role in the pathogenesis of NASH [12]. Indeed, the P2RX7^+^ cell pool comprised CD45^+^, CD14^+^, and CD68^+^ inflammatory cells (Figs 1B and S1A), and populations of cells expressing both CD45, CD14, or CD68 and P2RX7 increased by ~ 2.9-, ~ 6.9-, and ~ 10-fold, respectively, in NASH-affected livers compared to healthy control (Fig 1B). As P2RX7 drives both inflammasome activation and IL-1β processing and release [32–34], we examined inflammasome activation and mature IL-1β in NASH-affected liver biopsies. Levels of mature IL-1β (Fig 1C), caspase-1 (Fig 1D), *NLRP3*, *IL-1β*, *CASP1*, and *AIM2* mRNA (Fig 1G) and CCL2 and CCL5 chemokines (Fig 1E and 1F), key mediators of inflammatory cell recruitment, were also significantly elevated in NASH-affected liver biopsies compared to healthy controls, confirming earlier studies [14,15,35,36]. As expected, the levels of mRNA of genes encoding for inflammation (Fig 1H), HSC activation and fibrosis (S1B Fig), and ATP-dependent *ENTPD1* ectonucleotidase (S1C Fig), but not *NT5E* or ENTPD1 secreted levels (S1D Fig), were also increased in livers of patients with NASH (*vs* healthy controls). Collectively, these results suggest that the higher abundance of P2RX7^+^ cells in NASH-affected livers is associated with increased numbers of resident and infiltrating macrophages and increased inflammation and fibrosis.

### P2RX7 regulates the proinflammatory profile of MOs and KCs

**Fig 2:**
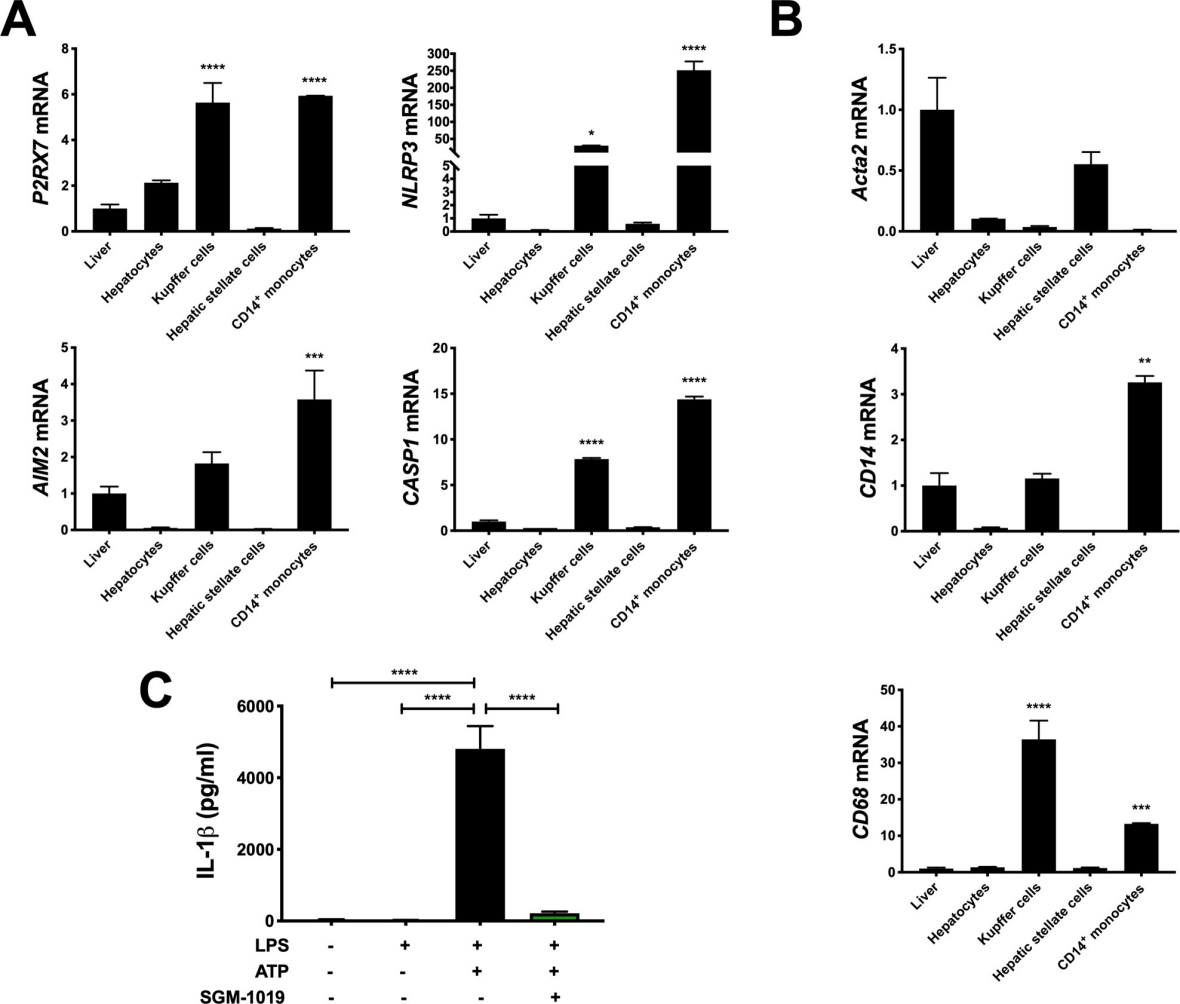
Inhibition of P2RX7 blocks IL-1β-induced inflammasome activation in MOs. (A) Relative expression of *P2RX*7 and NLRP3 inflammasome components *NLRP3*, *AIM2*, and *CASP1* in liver tissue and human primary hepatocytes, KCs, HSCs, and MOs. (B) Relative expression of *ACTA2*, *CD14*, and *CD68* in liver tissue and human primary hepatocytes, KCs, HSCs, and MOs. (C) IL-1β levels in culture media from MOs treated with LPS, ATP ± SGM-1019. In all statistical plots, the data are shown as the mean ± SEM. *P ≤ 0.05, **P ≤ 0.01, ***P ≤ 0.001, ****P ≤ 0.0001 by one-way ANOVA.

**Fig 3:**
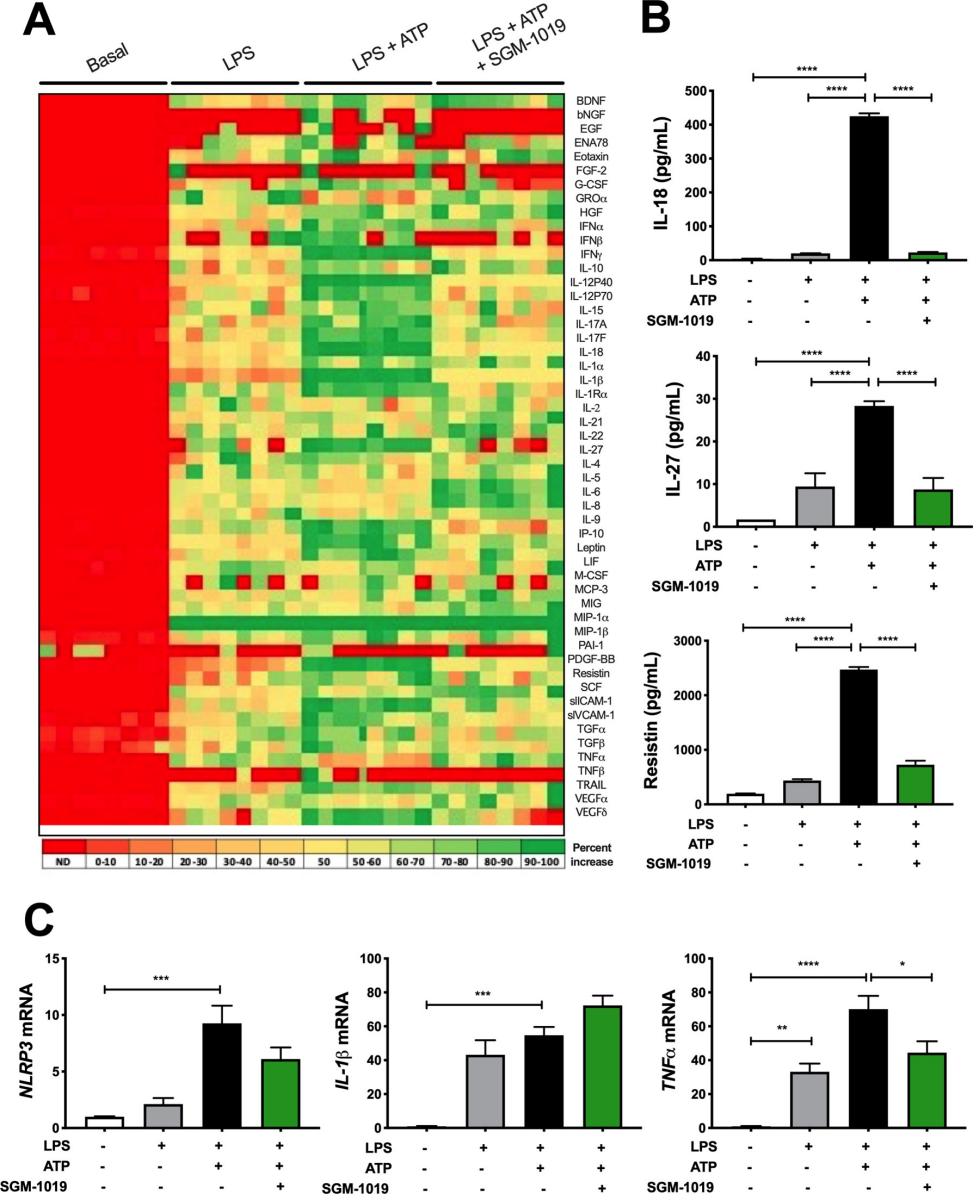
Inhibition of P2RX7 alters the secretion of cytokines in MOs. (A) Heat map showing levels of cytokines and growth factors present in the culture media from MOs treated with LPS, ATP ± SGM-1019. Red indicates low relative expression, and green indicates high relative expression. (B) IL-18, IL-27 and resistin levels in culture media from MOs treated with LPS, ATP ± SGM-1019. (C) Relative expression of *NLRP3*, *IL-1β*, and *TNFα* in MOs treated with LPS, ATP ± SGM-1019. In heat map, red indicates low relative expression, and green indicates high relative expression. n.d., for not detected. In all statistical plots, the data are shown as the mean ± SEM. *P ≤ 0.05, **P ≤ 0.01, ***P ≤ 0.001, ****P ≤ 0.0001 by one-way ANOVA.

We next investigated the role of P2RX7 and inflammasome activation in MOs and KCs. Consistent with our findings on liver biopsies (Figs 1 and S1), MOs and KCs expressed significantly greater levels of *P2RX7* and NLRP3 inflammasome components (*e*.*g*., *NLRP3*, *AIM2*, *CASP1*) mRNA than human liver, or isolated human primary hepatocytes and HSCs (Fig 2A). *ACTA2*, *CD14*, and *CD68* mRNA were expressed at highest levels in HSCs, MOs, and KCs, respectively (Fig 2B). Given that P2RX7 regulates IL-1β release in a variety of immune cells [37–43], and the high levels of expression of *P2RX7* and NLRP3 inflammasome components in MOs, we investigated IL-1β secretion in MOs and KCs. Indeed, lipopolysaccharide (LPS)-pretreated MOs stimulated with ATP released high levels of IL-1β (Fig 2C). IL-1β secretion from hepatocytes and HSCs was below detection levels (< 5 pg/ml). To evaluate the extent to which pharmacological inhibition of P2RX7 affects the regulation of IL-1β in MOs, a potent and selective P2RX7 inhibitor, SGM-1019 (also known as EVT-401) [44] [EC_95_ of 768 nM; IL-1β, human blood (S2A–S2C Fig)], was used to inhibit the activity of this receptor. Consistent with previous studies [39,42], P2RX7 inhibition with SGM-1019 blocked IL-1β released by LPS-pretreated MOs stimulated with ATP (Fig 2C). To further understand the role of P2RX7 regulating the inflammatory profile of MOs, we analyzed whether P2RX7 inhibition affects the secretion of multiple inflammatory cytokines and growth factors. Interestingly, similar to IL-1β, secretion of several cytokines and growth factors which were triggered by inflammasome activation were also reduced by SGM-1019 treatment in MOs (Fig 3A), including IL-18, IL-27, and resistin (Fig 3B) among others. *NLRP3*, *IL-1β*, and *TNFα* mRNA levels were significantly elevated in MOs stimulated with LPS and ATP (Fig 3C). Although no significant changes in *IL-1β* and *NLRP3* expression were observed as a result of P2RX7 inhibition (Fig 3C), *TNFα* expression was reduced significantly by SGM-1019 treatment. Altogether, these results indicate that P2RX7 not only regulates IL-1β levels, but also affects the levels of other cytokines secreted by MOs, and suggest that P2RX7 inhibition might contribute by reducing the proinflammatory profile of infiltrating macrophages in the liver.

**Fig 4:**
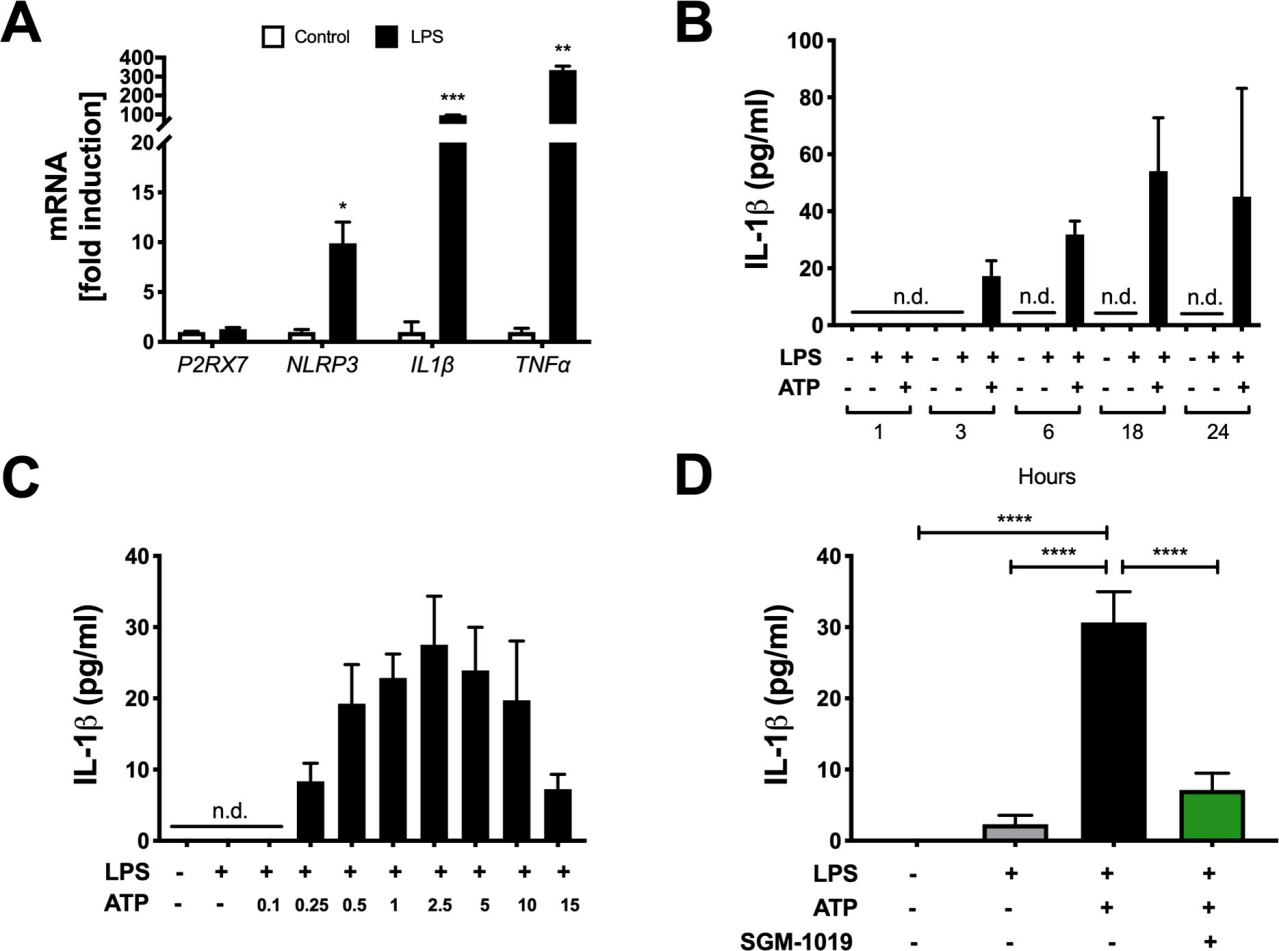
Inhibition of P2RX7 blocks IL-1β-induced inflammasome activation in KCs. (A) Relative expression of *P2RX*7, *NLRP3*, *IL-1β*, and and *TNFα* in KCs treated with LPS for 1 hour. (B) IL-1β levels in culture media from KCs pretreated at different times of LPS stimulation with and without ATP. (C) IL-1β levels in culture media from KCs pretreated with LPS and stimulated with increasing doses of ATP. (D) IL-1β levels in culture media from KCs treated with LPS, ATP ± SGM-1019. n.d., for not detected. In all statistical plots, the data are shown as the mean ± SEM. *P ≤ 0.05, **P ≤ 0.01, ***P ≤ 0.001, ****P ≤ 0.0001 by one-way ANOVA.

**Fig 5:**
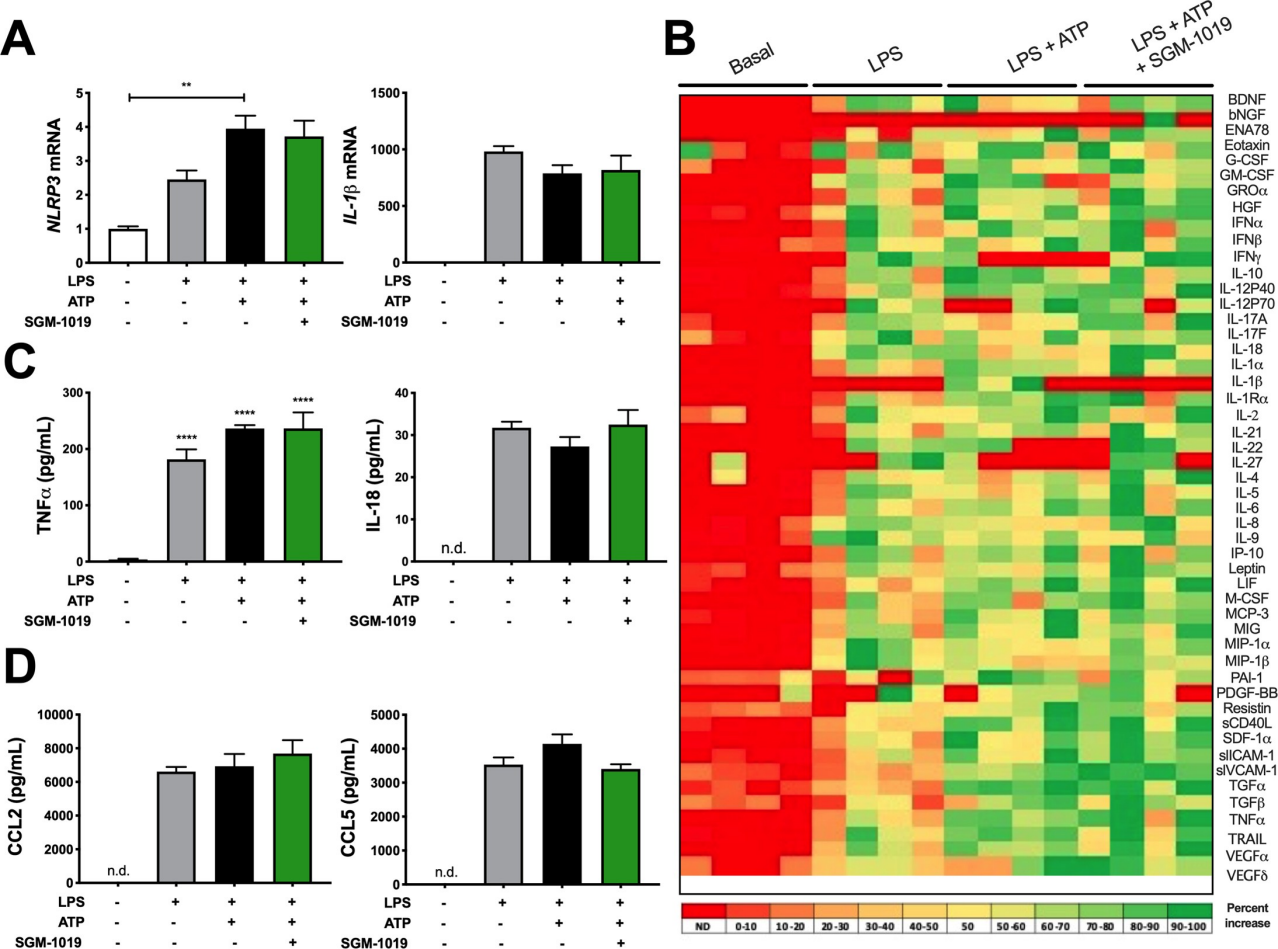
Inhibition of P2RX7 alters the secretion of cytokines in KCs. (A) Relative expression of *NLRP3* and *IL-1β*, (B) Heat map showing levels of cytokines and growth factors, (C) IL-18 and TNFα levels, and (D) CCL2 and CCL5 chemokine levels present in the culture media from KCs treated with LPS, ATP ± SGM-1019. In heat map, red indicates low relative expression, and green indicates high relative expression. n.d., for not detected. In all statistical plots, the data are shown as the mean ± SEM. *P ≤ 0.05, **P ≤ 0.01, ***P ≤ 0.001, ****P ≤ 0.0001 by one-way ANOVA.

Similar to the MOs (Fig 3C) and consistent with previous investigations [45], KCs exhibited a *NLRP3* and *IL-1β* transcriptional response to LPS (Fig 4A) and released IL-1β in maximum quantities following stimulation with LPS for 18 h (Fig 4B) and 2.5 mM of ATP for 45 min (Fig 4C). IL-1β secreted by KCs was also completely abrogated by SGM-1019 treatment (Fig 4D). However, the relative levels of this cytokine secreted by KCs were markedly lower (~ 100-fold) than those secreted by MOs (Figs 2C and 4D). Equally, no significant changes in *NLRP3* and *IL-1β* expression were observed as a result of P2RX7 inhibition by SGM-1019 in KCs (Fig 5A). To further investigate the role of P2RX7 regulating the inflammatory profile of KCs, multiple inflammatory cytokines and growth factors were also analyzed. Contrarily to MOs, secretion of cytokines and growth factors triggered by inflammasome activation in KCs (Fig 5B), including TNF*α* and IL-18 (Fig 5C), were not altered by SGM-1019 treatment. Furthermore, CCL2 and CCL5 secretion was increased in KCs treated with LPS but remained unaffected by addition of ATP or SGM-1019 (Fig 5D). In addition, ATP-dependent *ENTPD1* ectonucleotidase gene expression was not altered in MO and KC treated with LPS and/or ATP (S3A and S3B Fig) and ENTPD1 secretion levels was below detection levels (< 40 pg/ml). Collectively, these results show that human primary MOs and KCs serve as a major source of IL-1β in the livers, that P2RX7 plays an integral role in modulating IL-1β secretion in these cells, and that P2RX7 inhibition differently affects the proinflammatory profile of resident and infiltrating macrophages.

### P2RX7-mediated IL-1β contributes to hepatocyte death and chemokine secretion in hepatocytes

**Fig 6:**
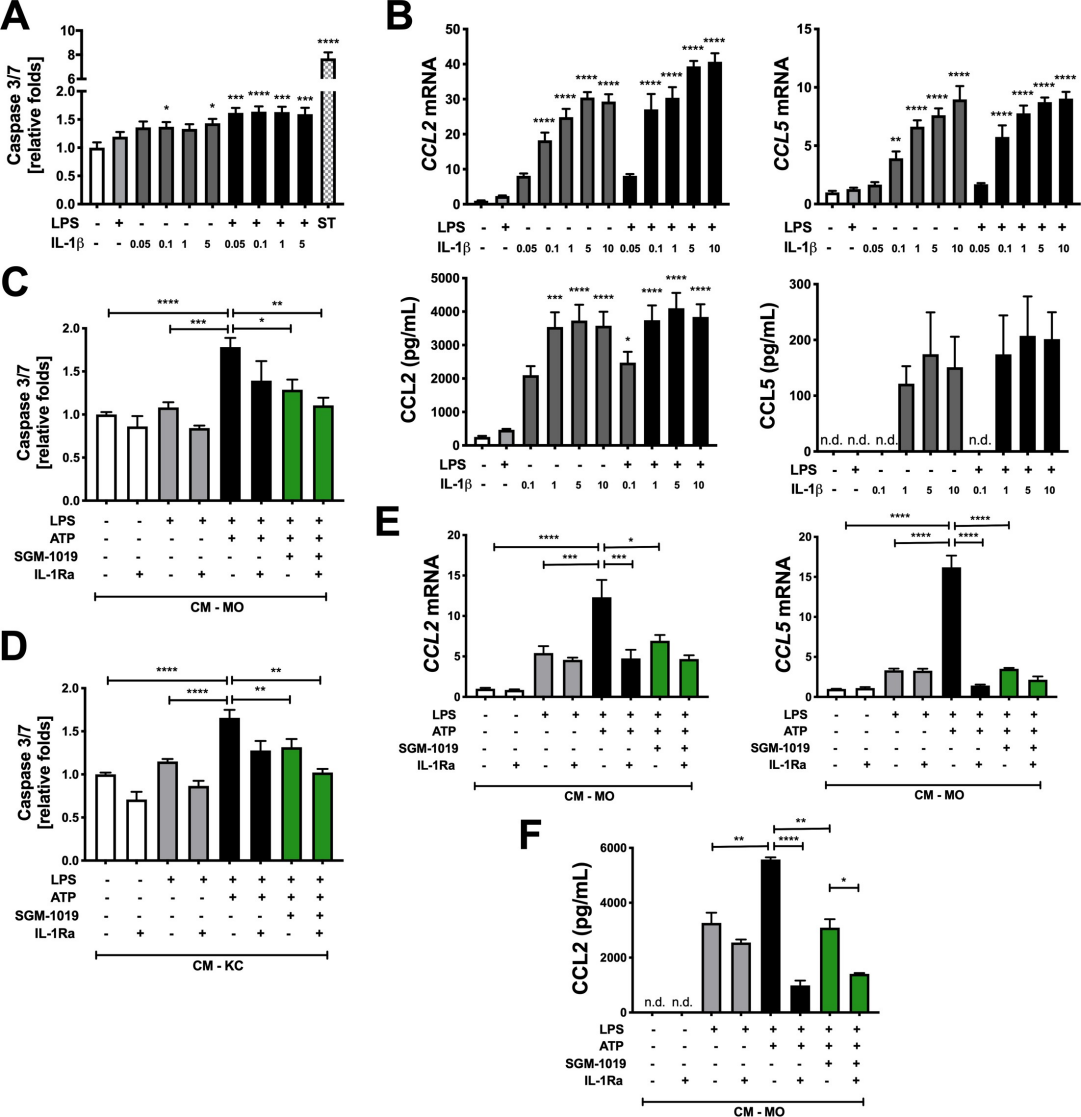
P2RX7-mediated IL-1β modulates hepatocyte death and CCL2/CCL5 expression and secretion in hepatocytes. (A) Relative caspase 3/7 activity in human primary hepatocytes treated with LPS ± increasing concentrations (in ng/ml) of recombinant human IL-1β (IL-1β). Staurosporine (ST) was used as a positive control. (B) Relative expression of *CCL2*/*CCL5* and CCL2/CCL5 levels in human primary hepatocytes treated with LPS ± increasing concentrations of IL-1β. Relative caspase 3/7 activity in hepatocytes cultured with (C) CM-MO or (D) CM-KC treated with LPS, ATP ± SGM-1019 and/or IL-1Ra. (E) Relative expression of *CCL2*/*CCL5* and (**F**) CCL2 levels in human primary hepatocytes cultured with CM-MO treated with LPS, ATP ± SGM-1019 and/or IL-1Ra. n.d., for not detected. In all statistical plots, the data are shown as the mean ± SEM. *P ≤ 0.05, **P ≤ 0.01, ***P ≤ 0.001, ****P ≤ 0.0001 by one-way ANOVA.

IL-1β plays a key role in the pathogenesis of liver injury [20–22,45]. Although recent studies have implicated IL-1β in CCL2 secretion and lipid accumulation in murine hepatocytes [23,46] and increased apoptosis in hepatocytes isolated from choline-deficient L-aminoacid-defined (CDAA)-fed mice [46], little is known about the implication of P2RX7. As such, we conducted experiments to determine whether exogenous IL-1β and P2RX7-dependent IL-1β released by human primary MOs and KCs regulate hepatocyte apoptosis and chemokine secretion. While caspase 3/7 activity increased in human primary hepatocytes upon treatment with recombinant human IL-1β (Fig 6A), the addition of LPS greatly enhanced the effect of this cytokine and amplified caspase 3/7 activity (Fig 6A). In addition, IL-1β increased *CCL2* and *CCL5* expression and the secretion of these chemokines in hepatocytes (Fig 6B). Contrary to the trend observed with apoptosis, however, LPS exhibited little to no influence on the impact of IL-1β on chemokines production (Fig 6B). Since IL-1β induces ER stress in pancreatic epithelial cells [47] and hepatocyte endoplasmic reticulum (ER) stress induces NLRP3 inflammasome activation and hepatocyte death [48,49], we examined whether IL-1β affected ER stress in human primary hepatocytes. Intriguingly, exogenous IL-1β increased the expression of numerous ER stress genes (*e*.*g*., *DR5*, *DDIT3*, *ERO1α* and *GADD34*) in hepatocytes, and the addition of LPS enhanced the effect of IL-1β (S4A Fig).

To determine whether IL-1β secreted by MOs and KCs regulates hepatocyte apoptosis, CCL2/CCL5 secretion, and ER stress in a P2RX7-dependent manner, human primary hepatocytes were cultured with conditioned media (CM) originating from MOs (CM-MO) or KCs (CM-KC) treated with LPS and ATP ± SGM-1019 (Figs 2C and 4D). While caspase 3/7 activity significantly increased in hepatocytes cultured with CM-MO or CM-KC challenged with LPS and ATP, the very same CM with SGM-1019 treatment resulted in significantly reduced P2RX7-mediated caspase 3/7 activity in hepatocytes (Fig 6C and 6D). No changes were observed in caspase-1 expression (S4B Fig) and hepatocyte viability (S4C Fig). However, hepatocytes pretreated for 1 hour and cultured with the same CM in the presence of 100 ng/ml of IL-1 receptor antagonist (IL-1Ra) did not significantly reduce caspase 3/7 activity (Fig 6C and 6D) or increase hepatocyte viability (S4C Fig). Furthermore, human primary hepatocytes cultured with CM-MO challenged with LPS and ATP induced *CCL2* and *CCL5* expression and CCL2 secretion, and same CM with SGM-1019 or IL-1Ra treatment of hepatocytes significantly reduced the effects mediated by P2RX7 (Fig 6E and 6F). In contrast to hepatocytes cultured with CM-MO, hepatocytes cultured with CM-KC challenged with LPS and/or ATP did not show P2RX7-induced *CCL2* and *CCL5* gene expression (S4D and S4E Fig). Expression of the ER stress gene *GADD34* was increased with CM-MO and CM-KC challenged with LPS and ATP (S4F and S4G Fig), but culturing hepatocytes with the same CM + SGM-1019 did not reduce it (S4F and S4G Fig), suggesting no effect of P2RX7-dependent IL-1β on ER stress in hepatocytes. Expression of *DR5*, *DDIT3*, and *ERO1α* were not affected by CM-MO or CM-KC (S5A–S5C Fig). These results suggest that P2RX7-dependent IL-1β contributes to hepatocyte death and chemokine secretion and that P2RX7-induced inflammatory profiles in MOs and KCs differentially affects hepatocyte responses.

### P2RX7-mediated IL-1β enhances procollagen and chemokine secretion in HSCs

**Fig 7:**
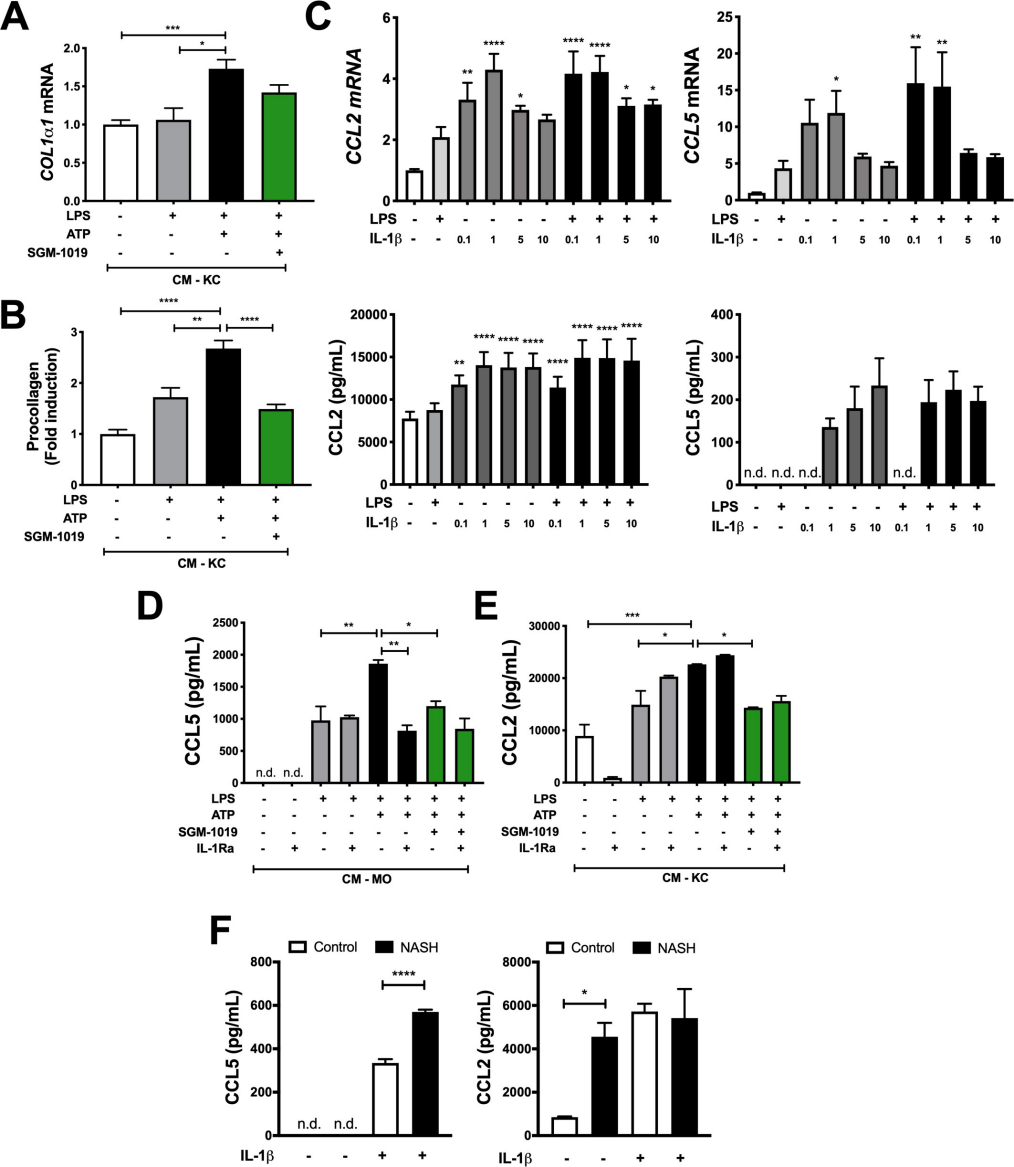
P2RX7-mediated IL-1β enhances procollagen and CCL2/CCL5 chemokine secretion in HSCs. (A) Relative expression of *Col1α1* and (B) procollagen levels in HSCs cultured with CM-KC treated with LPS, ATP ± SGM-1019 (CM-KC). (C) Relative expression of *CCL2*/*CCL5* and CCL2/CCL5 levels in culture media from HSCs treated with LPS ± increasing concentrations of IL-1β. (D) CCL5 and (E) CCL2 levels in HSCs cultured with CM-MOs or CM-KCs treated with LPS, ATP ± SGM-1019 and/or IL-1Ra, respectively. (F) CCL5 and CCL2 levels in HSCs isolated from control and NASH-affected donor treated with IL-1β (*n* = 2 individuals per group) *P ≤ 0.05, ****P ≤ 0.0001 by unpaired two-sided Student’s t-tests. n.d., for not detected. In all statistical plots, the data are shown as the mean ± SEM. n.s, for not significant. *P ≤ 0.05, **P ≤ 0.01, ***P ≤ 0.001, ****P ≤ 0.0001 by one-way ANOVA.

As IL-1β is known to promote the activation of murine HSCs [46], we assessed the effects of exogenous IL-1β and P2RX7-dependent IL-1β released by MOs and KCs on HSCs activation and fibrosis. Consistent with previous reports [46], exogenous IL-1β increased *COL1α1* expression in HSCs, and the addition of LPS enhanced this effect (S6A Fig). To determine whether MO- and/or KC-derived IL-1β regulate HSC activation in a P2RX7-dependent manner, HSC were cultured with CM-MO and CM-KC challenged with LPS and ATP ± SGM-1019 (Figs 2C and 4D). While CM-MO did not alter *COL1α1* or *COL4α*1 gene expression in HSCs (S6B Fig), CM-KC challenged with LPS and ATP significantly increased *COL1α1*, *COL4α1*, and *COL1α2* expression in HSCs (Figs 7A and S6C). However, the addition of SGM-1019 did not significantly reduce fibrotic gene expression (Figs 7A and S6C). Interestingly, procollagen type I secretion also increased significantly in HSCs cultured with CM-KC treated with LPS and ATP (Fig 7B), but diminished significantly in the presence of SGM-1019 (Fig 7B). Procollagen secretion was not altered in HSCs cultured with CM from MOs (below detection levels, < 5 pg/ml). These results suggest that P2RX7-dependent IL-1β released by KCs regulates fibrosis by modulating HSC procollagen secretion.

As murine HSCs express *CCL2* and *CCL5* and secrete their gene products [50,51], experiments were conducted to determine whether exogenous IL-1β and human primary MO- and/or KC-derived IL-1β regulate human primary HSC chemokine secretion. Exogenous IL-1β increased *CCL2* and *CCL5* expression and secretion of their gene products in HSC (Fig 7C). In contrast to the results of P2RX7-mediated chemokines in hepatocytes, secretion of CCL5 and CCL2 by HSC was significantly increased by CM-MO and CM-KC challenged with LPS and ATP, respectively (Fig 7D and 7E). The same CM containing SGM-1019 resulted in significantly reduced CCL5 and CCL2 secretion (Fig 7D and 7E), while treatment with IL-1Ra only altered CCL5 secretion from HSC cultured with CM-MO challenged with LPS and ATP. No significant changes were observed in *CCL2* and *CCL5* gene expression in HSCs cultured with CM-MO and CM-KC challenged with LPS, ATP and SGM-1019 or treated with IL-1Ra (S7A and S7B Fig) and in CCL2 and CCL5 secretion by HSCs cultured with CM-MO and CM-KC, respectively (S7C Fig). To further examine the effects of IL-1β on HSCs chemokine secretion, HSCs from patients with NASH and healthy control livers were isolated and treated with exogenous IL-1β. Interestingly, IL-1β significantly increased CCL5 secretion in HSC from NASH-affected liver compared to control (Fig 7F). On the contrary, CCL2 showed no differences after IL-1β treatment (Fig 7F), but its basal secretion in HSCs from NASH-affected liver was significantly increased compared to control (Fig 7F), suggesting that HSCs from NASH-affected livers are already sensitized for CCL2 secretion. Overall, these results demonstrate that P2RX7-dependent IL-1β contributes to CCL2, CCL5, and procollagen secretion in HSCs and P2RX7-induced inflammatory profile in MOs and KCs differentially affects HSCs.

### Pharmacological inhibition of P2RX7 protects against inflammation and fibrosis in a liver fibrosis model in non-human primates

**Fig 8:**
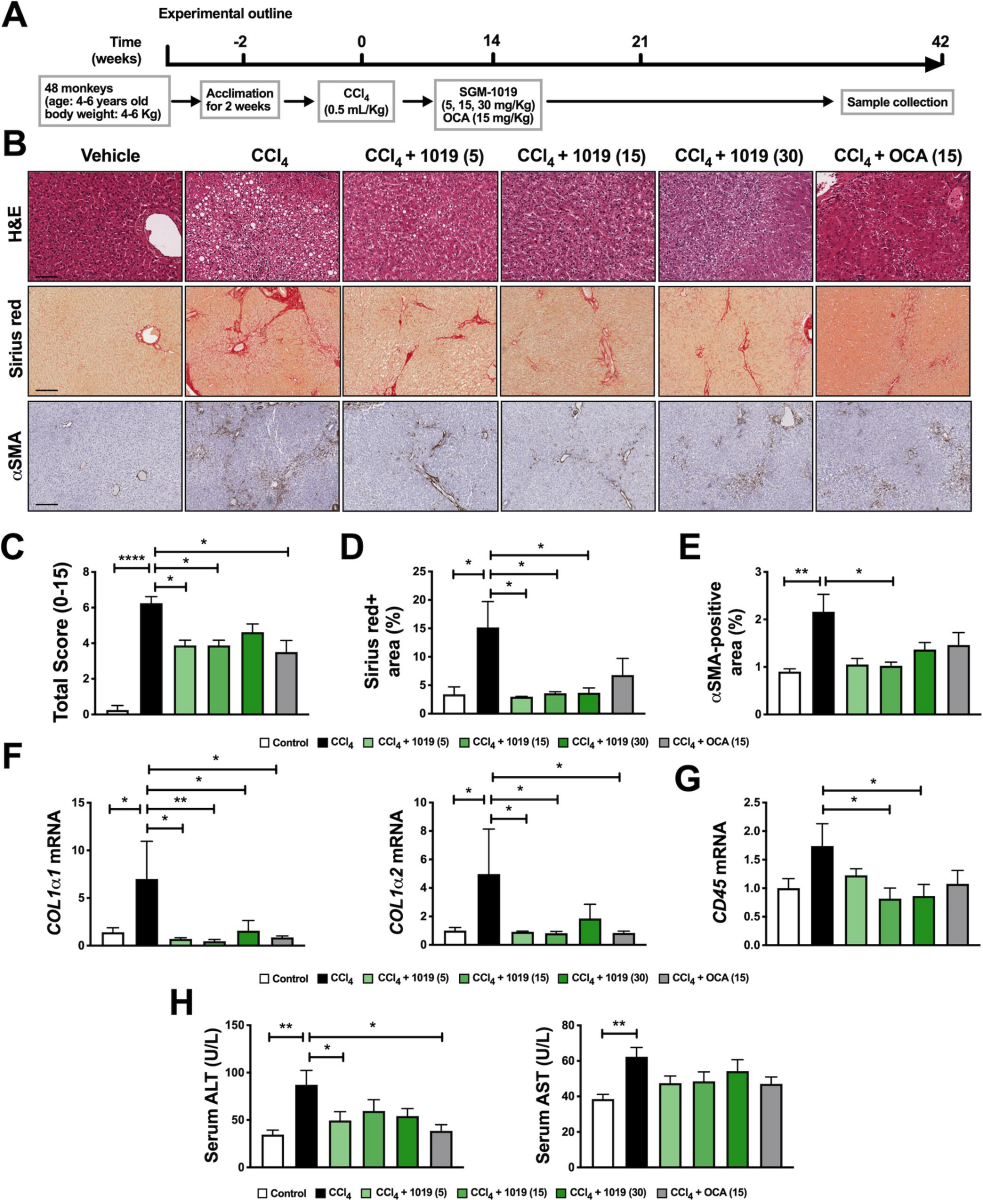
Pharmacological inhibition of P2RX7 protects against inflammation and fibrosis in a CCl_4_-induced liver fibrosis model in non-human primates. (A) Schematic representation of the timing strategy used to evaluate the effects of pharmacological inhibition of P2RX7 with SGM-1019 in CCl_4_-induced liver fibrosis in monkeys. (B) Representative images of H&E (objective 20X), Sirius red-stained and αSMA (objective 10X) expression determined by immunohistochemistry in liver sections from monkeys. Scale bar, 50 μM for H&E and 100 μM for Sirius red and αSMA. (C) Total histological score from liver sections. (D) Percentage of Sirius red staining area. (E) Quantification of αSMA expression in liver sections. (F) Relative expression of *COL1*α1 and *COL1*α2, and (**G**) *CD45* in the livers from monkeys. (H) Serum levels of ALT and AST in monkeys after treatments. In all statistical plots, the data are shown as the mean ± SEM. *P ≤ 0.05, **P ≤ 0.01, ***P ≤ 0.001, ****P ≤ 0.0001 by one-way ANOVA.

To evaluate the relevance of our *in vitro* findings and to study the potential therapeutic effect of pharmacologically inhibiting P2RX7 in non-human primates, a more clinically translatable animal model. Liver fibrosis in cynomolgus monkeys (*Macaca fascicularis*) was induced by CCl_4._ Although the CCl_4_ model of fibrosis is different from NASH, it shares common pathways of hepatocyte injury, inflammation, and fibrosis through HSC activation. Once acclimated, monkeys were randomly divided into six distinct groups. The subjects in five of these groups received CCl_4_ injections for six weeks to induce liver fibrosis. The monkeys in three of these groups were administered SGM-1019 in varying doses (5, 15, and 30 mg/Kg) or a single dose of obeticholic acid (OCA) starting at week 2 (Fig 8A). OCA has recently been shown to have antifibrotic effects in patients [52], and was used as a positive control. Corroborating previous work [53], histological analyses of liver sections from monkeys treated with CCl_4_ showed significantly increased liver pathology including ballooning degeneration, inflammation, and fibrosis (Fig 8B). Liver sections from monkeys administered CCl_4_ and SGM-1019 exhibited remarkably improved liver pathology (Fig 8B).

Comprehensive scoring (combination of ballooning degeneration, steatosis, inflammation, and fibrosis scores) revealed significant histological improvements in the livers of monkeys administered with SGM-1019, as well as OCA (Fig 8C), without significant changes in body or liver weight (S8A and S8B Fig). Consistent with reduced fibrosis (Fig 8B) and collagen deposition (Fig 8D), immunohistochemistry for α-smooth muscle actin (αSMA), a marker of hepatic stellate cell activation, was significantly reduced by SGM-1019-mediated P2RX7 inhibition (compared to CCl_4_ vehicle; Fig 8B and 8E). The expression of profibrotic genes and inflammatory markers were significantly (Fig 8F and 8G) or moderately (S8C and S8D Fig) reduced in CCl_4_ with SGM-1019-treated monkeys, compared to their vehicle counterparts. Serum ALT concentrations were significantly reduced and AST concentrations were marginally reduced following SGM-1019 administration compared to vehicle control (Fig 8H). These results show that P2RX7 inhibition partially ameliorates the hepatotoxicity of CCl_4_. Taken together, our results show that pharmacological inhibition of P2RX7 results in amelioration of liver pathology in non-human primates and supports the anti-inflammatory and anti-fibrotic effects of P2RX7 inhibition observed *in vitro*.

## Discussion

While lifestyle intervention and weight loss are the only therapeutic options for the management of NASH [6], multiple agents are in various phases of clinical development for its treatment [54]. Amongst them, a limited number directly target inflammatory pathways or partly act through effects on them, resulting in moderated improvements in inflammation, and fibrosis [55]. These findings have provided a strong rationale for targeting inflammatory pathways in NASH. As such, previous studies have showed that P2RX7 mediates liver injury [56], oxidative stress [29], fibrosis [57], and autophagy [28] in several diet- and chemically-induced liver injury murine models. Furthermore, P2RX7 is a major driver of NLRP3 inflammasome activation and IL-1β processing [24], which are two major contributors to hepatocyte damage, immune cell activation, and amplification of liver inflammation [8,58]. Taken collectively, these attributes have rendered P2RX7 an attractive target for liver diseases, including NASH. Our study reveals that P2RX7 and NLRP3 inflammasome components, caspase-1, and mature IL-1β are enriched in livers of NASH-affected subjects. Our work also shows that the increased number of P2RX7 positive cells results primarily from the activation of resident CD68^+^ KCs and CD14^+^ infiltrating macrophages.

Hepatic inflammation, including macrophage infiltration and KC activation, is a hallmark characteristic of NASH pathology [9] and P2RX7 is readily expressed by MOs and macrophages [37,59–61]. Since other liver resident cells are also known to express P2RX7 (*e*.*g*., hepatocytes, HSC) [62], we cannot exclude the potential P2RX7 contribution from other such sources. However, our findings demonstrate that MOs and KCs express *P2RX7* and NLRP3 inflammasome components at much higher levels than their hepatocytes and HSCs counterparts. Moreover, whereas P2RX7 regulated IL-1β secretion in both human primary MOs and KCs challenged with LPS and ATP, two required signals to activate P2RX7 and inflammasome, mature IL-1β was not detected in HSCs and hepatocytes. Therefore, these results implicate infiltrating and resident macrophages as the major contributors of increased P2RX7, NLRP3 inflammasome and IL-1β in NASH-affected livers.

In cell-autonomous systems, both MOs, and KCs secrete IL-1β in response to being challenged with LPS + ATP. Consistent with the findings of previous investigations [39,42], P2RX7 inhibition completely blocked IL-1β secretion, indicating the necessity of P2RX7 for mature IL-1β processing in these cells. While P2RX7 inhibition in LPS + ATP-challenged KCs exclusively reduced IL-1β, P2RX7 inhibition in LPS + ATP-challenged MOs not only reduced IL-1β but others such as IL-18, IL-27, and resistin among others. Interestingly, elevated levels of resistin have previously been reported in NASH patients [63,64], and IL-27 is known to inhibit T cell responses [65], suggesting that P2RX7 inhibition may reduce the proinflammatory response of not only MOs but also other liver immune cells. These results also suggest that ATP signaling via P2RX7 regulates a variety of different cytokines in MOs, but the determination of its precise mechanism requires further investigation. However, it is possible that cultured MOs simply respond differently than infiltrating MOs in NASH-affected livers or that P2RX7 inhibition could also have undesired effects by altering the inflammatory response of infiltrating MOs. The diversity and heterogeneity of macrophages in liver diseases [66] underscore the importance of P2RX7 and the need to better understand the roles of this receptor in liver-specific macrophage functions.

ATP-induced ectonucleotidases such as ENTPD1 (also known as CD39) regulate P2RX7 by generating AMP from extracellular ATP limiting P2RX7 activation and pro-inflammatory responses. Inhibition of P2RX7 in LPS-primed murine macrophages attenuates cytokine production and prevents ATP-induced increases in ENTPD1 [67,68]. Interestingly, although our data shows that ENTPD1 secreted levels were not significantly increased, *ENTPD1* gene expression was increased in NASH-affected livers compared to controls, suggesting a potential role of ectonucleotidases during NASH. However, *ENTPD1* gene expression was not altered in in LPS + ATP-challenged MO or KC. These results suggest that other cell type/s might be responsible for *ENTPD1* expression in livers with NASH, or, more likely, that the treatment of human primary KC and CD14^+^ monocytes require different times and/or concentrations of LPS and ATP treatment to potentially induce ENTPD1. In this regard, it is also likely that different concentrations of LPS are needed to induce mouse peritoneal or bone marrow-derived macrophages and human CD14^+^ monocytes responses. Future studies will address the potential role of ectonucleotidases and their role in NASH progression.

Activated KCs, HSCs, and injured hepatocytes all secrete high levels of CCL2 [46,69,70], which exacerbates liver fibrosis by mediating the recruitment of inflammatory cells to the site of liver injury [71–76]. Similarly, CCL5 levels are upregulated in the livers of patients with fibrosis [72] and HSCs are considered to be an important source [77]. In our study, P2RX7-dependent IL-1β secretion by MOs and KCs regulated CCL2 and CCL5 chemokines in hepatocytes and HSCs. Blockade of IL-1β secretion by P2RX7 inhibition in MOs reduced *CCL2*/*CCL5* gene expression and CCL2 secretion in hepatocytes, and CCL5 secretion in HSCs. In contrast, blockade of IL-1β secretion by P2RX7 inhibition in KCs reduced *CCL5* gene expression in hepatocytes and CCL2 secretion in HSCs. In addition, incubation of hepatocytes and HSCs with IL-1Ra markedly reduced CCL2 and CCL5 gene expression and secretion in hepatocytes and CCL5 secretion in HSCs, confirming IL-1β as a critical mediator for CCL2 and CCL5 secretion by hepatocytes and HSCs. These results are in agreement with the findings in a previous study that reported IL-1β induction of CCL2 secretion in murine hepatocytes [46]. The distinct effects on CCL2 and CCL5 gene expression and secretion observed between hepatocytes and HSCs cultured with CM from MOs and KCs might be explained by the lower levels of IL-1β secreted by KCs compared to MOs. In this regard, we cannot also exclude that diverse and distinct levels of additional secreted cytokines between MOs and KCs that might be also affecting CCL2 and CCL5 gene expression and secretion in hepatocytes and HSCs. We show that blockade of IL-1β secretion by P2RX7 inhibition in MOs and KCs reduces hepatocyte apoptosis. However, incubating hepatocytes with IL-1Ra in culture media from MOs did not affect the activities of caspase 3/7 or caspase-1. As such, additional mechanisms of P2RX7 and/or secreted factors from MOs are likely involved in the regulation of hepatocyte cell death.

In addition, future studies will address the effects of lipotoxicity in the liver, a key step in NASH progression, by studying how fat accumulation and high levels of different saturated and unsaturated fatty acids in the liver may affect P2RX7 signaling pathway and altering homeostasis in different liver resident cells. In this regard, palmitic acid and LPS are known to trigger NLRP3 activation and IL-1β in hepatocytes [14], and free-fatty acids, cholesterol and lipid metabolites have been shown to affect a variety of biological functions in resident and infiltrating macrophages [12], adding complexity in the mechanisms and signals between these different cells. Therefore, it is necessary to better understand the role of fatty acids on the P2RX7 signaling pathway and its effects not only on hepatocyte cell death and chemokine secretion but also resident and infiltrating macrophages cytokine secretion and hepatic stellate cell differentiation and fibrogenesis.

Our results also show that exogenous IL-1β and CM from LPS + ATP-challenged KCs induce fibrogenesis in human primary HSCs. Consistent with these results, previous studies demonstrated that IL-1β promotes the activation of murine HSCs, by increasing mRNA and protein expression of TIMP-1, and mRNA of *COL1α1*, *COL4α1*, and *PAI-1*, but not in IL-1R^-/-^ HSCs [46]. Indeed, IL-1β plays an important role in fibrogenic responses in several diet-induced liver injury murine models [19,21,23,46]. However, neither CM from LPS + ATP-challenged MOs nor P2RX7 inhibition in LPS + ATP-challenged KCs altered the expression of fibrotic genes in HSCs, suggesting that P2RX7 inhibition is not sufficient to reduce fibrotic gene expression and/or additional cytokines secreted by MOs and KCs are influencing the different responses to IL-1β. Interestingly, P2RX7 inhibition in LPS + ATP-challenged KCs reduced procollagen secretion. Future studies will explore the contribution of P2RX7-dependent IL-1β in the formation and regulation of collagen by HSCs in NASH.

**Fig 9:**
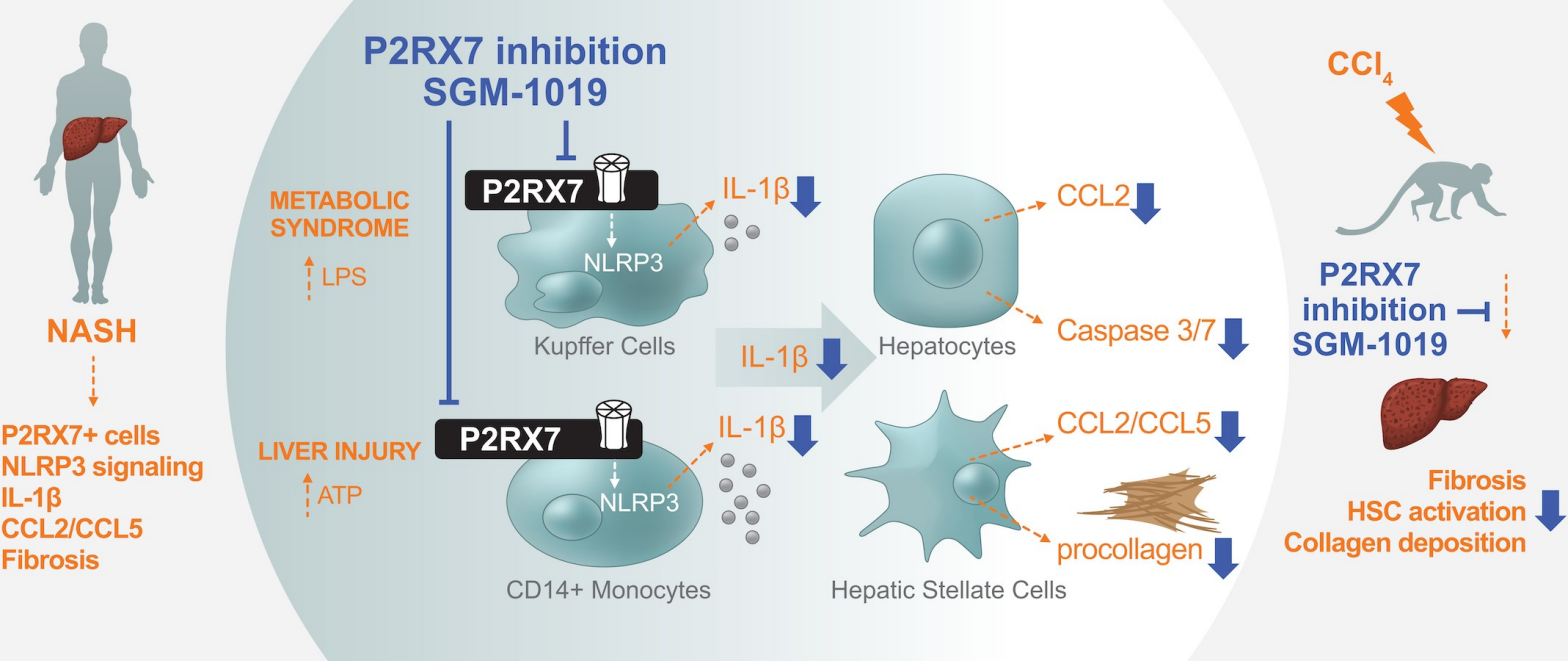
Schematic summary of P2RX7 in NASH-affected livers and effects of P2RX7 pharmacological inhibition *in vitro* and *in vivo*. NASH-affected livers show greater number of cells expressing P2RX7, NLRP3 inflammasome activation, IL-1β, CCL2/CCL5 and fibrosis. P2RX7 is expressed by infiltrating MOs and resident KCs in the livers of NASH-affected individuals. Pharmacological inhibition of P2RX7 in human primary CD14^+^ MOs and KCs block IL-1β release and differentially modulates their inflammatory response. Reduced P2RX7-dependent IL-1β secretion from MOs and KCs results in decreased hepatocyte damage, chemokine secretion, and HSCs fibrosis. P2RX7 pharmacological inhibition in a more clinically translatable animal model results in significant protection from inflammation and fibrosis.

Inhibition of P2RX7 yielded anti-inflammatory and anti-fibrotic effects in a CCl_4_-induced liver fibrosis model in non-human primates. Livers from non-human primates treated with SGM-1019 exhibited significant histological improvements, showed reduced HSC activation, collagen deposition, fibrosis-related gene expression and improvements in serum ALT levels. The lack of an apparent dose response might be explained by the fact that plasma SGM-1019 levels were sustained at 95% receptor occupancy at all doses evaluated: for > 8 hours at the lowest dose of SGM-1019 (5 mg/Kg dose group; 8 hours, 630 ± 452 ng/ml; 12 hours, 316 ± 233 ng/ml), while at higher doses, receptor occupancy was above 95% for the whole duration of the study (15 mg/Kg dose group; 12 hours, 664 ± 406 ng/ml and 30 mg/Kg dose group; 12 hours, 1071 ± 761 ng/ml). The results obtained from non-human primates highlight the novel anti-fibrotic effects of P2RX7 inhibitors in an *in vivo* model, and support the notion of exploring their therapeutic effects clinically. However, it is important to highlight that the limited number of primates per study cohort constrained further analyses. Evidence from NASH clinical trials of early frontrunner drugs show that only 40% of patients benefit from a single therapy [54]. These results have prompted studies on combination therapies capitalizing on multiple mechanisms of action. As such, future studies will determine the efficacy of P2RX7 inhibitors in NASH patients, both as a stand-alone treatment and in concert with agents targeting pathways that affect other pathological aspects of NASH such as obeticholic acid (Farnesoid X receptor agonist), pioglitazone (peroxisome proliferator-activated receptor (PPAR)-γ agonist) and elafibranor (PPAR-α/δ agonist). In summary, we identified P2RX7 as a key signaling pathway for inflammation and fibrosis in liver injury by modulating the inflammatory response of infiltrating MOs and resident KCs which affect chemokine secretion and fibrosis (Fig 9). In conclusion, our study provides a strong rational to further evaluate P2RX7 inhibitors as a treatment for NASH.

## Supporting information

S1 Table Histological scores from healthy and NASH-affected liver biopsies from donors used in this study. (PDF)

S1 Fig NASH-affected liver biopsies show increased P2RX7^+^ cells, fibrogenesis, and ATP-dependent ectonucleotidases. (A) Representative images (objective 40X) of immunohistochemical staining of P2RX7 (brown) with CD45, CD14, or CD68 (red) in liver tissue from a representative control and NASH donor. Scale bar, 100 μM. Black arrows highlight P2RX7^+^ cells and black arrowheads highlight CD45^+^, CD14^+^, and CD68^+^ cells. Area in square is shown amplified on right column images. (B) Relative expression levels of *COL1α1*, *COL1α2*, *COL4α1*, *ACTA2* and *TGFβ* in liver tissue from control and NASH donors (*n* = 5 individuals per group). (C) Relative expression levels of *ENTPD1* and *NT5E* and (D) ENTPD1 levels in liver tissue from control and NASH donors (*n* = 5 individuals per group). In all statistical plots, the data are shown as the mean ± SEM. *P ≤ 0.05, **P ≤ 0.01, ***P ≤ 0.001, ****P ≤ 0.0001 by two-sided Student’s t-test.
(TIFF)

S2 Fig SGM-1019 is a potent P2RX7 antagonist that blocks ATP-mediated IL-1β production in whole blood of primates and humans. (A) Species comparison of SGM-1019 in an *ex vivo* whole blood ATP-dependent IL-1β release assay. Table shows inhibitory concentration (IC) of SGM-1019 at 50% (IC_50_), 95% (IC_95_), and maximum percent inhibition (MPI) of SGM-1019 in human and primate blood. AUC = Area under curve. Data in nanomolar (nM) is shown as the mean ± SEM (human) and individual replicates (NHP). Effect of SGM-1019 (0–3 μM) on LPS/ATP induced IL-1β secretion expressed as a proportion of IL-1β secretion of vehicle treated LPS/ATP primed blood (E/E_max_) in (B) non-human primate (*n* = 2 monkeys) and (C) human (*n* = 3 individuals). Blood was treated with LPS plus SGM-1019 for 1 hour (human) or 2 hours (NHP) prior to the addition of ATP (0–20 mM) for 45 min.
(TIFF)

S3 Fig ATP-dependent ENTPD1 ectonucleotidase gene expression is not altrered in human CD14^+^ monocytes and KC treated LPS and ATP. Relative expression of *ENTPD1* in (A) KC and (B) CD14^+^ monocytes treated with LPS, ATP ± SGM-1019. In all statistical plots, the data are shown as the mean ± SEM. n.s, for not significant. *P ≤ 0.05, **P ≤ 0.01, ***P ≤ 0.001, ****P ≤ 0.0001 by one-way ANOVA.
(TIFF)

S4 Fig Recombinant human and P2RX7-mediated IL-1β alters ER stress and induces *Ccl2*/*Ccl5* chemokine expression. (A) Relative expression of ER stress genes *DR5*, *DDIT3*, *ERO1α*, and *GADD34* in human primary hepatocytes treated with LPS (100 ng/ml) with or without increasing concentrations of IL-1β. (B) Caspase-1 and (C) Percentage of viability of human primary hepatocytes cultured with CM from CD14^+^ monocytes (CM-MO) treated with LPS, ATP ± SGM-1019 and/or IL-1Ra. Relative expression of (D) *CCL2* and (E) *CCL5* in hepatocytes cultured with CM from Kupffer cells (CM-KC) treated with LPS, ATP ± SGM-1019 and/or IL-1Ra. Relative expression of *GADD34* in human primary hepatocytes cultured with CM from (F) CD14^+^ monocytes (CM-MO) and (G) Kupffer cells (CM-KC) treated with LPS, ATP ± SGM-1019 and/or IL-1Ra. In all statistical plots, the data are shown as the mean ± SEM. n.s, for not significant. *P ≤ 0.05, **P ≤ 0.01, ***P ≤ 0.001, ****P ≤ 0.0001 by one-way ANOVA.
(TIFF)

S5 Fig P2RX7-mediated IL-1β does not regulate ER stress gene expression in hepatocytes. Relative expression of ER stress genes (A) *DR5*, (B) *DDIT3*, and (C) *ERO1α* in human primary hepatocytes cultured with CM from CD14^+^ monocytes (CM-MO) or with CM from Kupffer cells (CM-KC) both treated with LPS, ATP ± SGM-1019 and/or IL-1Ra. In all statistical plots, the data are shown as the mean ± SEM. n.s, for not significant. *P ≤ 0.05, **P ≤ 0.01, ***P ≤ 0.001, ****P ≤ 0.0001 by one-way ANOVA.
(TIFF)

S6 Fig P2RX7-mediated IL-1β does not regulate fibrotic gene expression in HSCs. (A) Relative expression of *COL1α1* in HSC treated with LPS ± increasing concentrations of rhIL-1β (IL-1β). (B) Relative expression of *COL1α1* and *COL4α1* in HSC cultured with conditioned media from CD14^+^ monocytes treated with LPS, ATP ± SGM-1019 (CM-KC). (C) Relative expression of *COL1α2* and *COL4α1* in HSC cultured with conditioned media from Kupffer cells treated with LPS, ATP ± SGM-1019 (CM-KC). In all statistical plots, the data are shown as the mean ± SEM. n.s, for not significant. *P ≤ 0.05, **P ≤ 0.01, ***P ≤ 0.001, ****P ≤ 0.0001 by one-way ANOVA.
(TIFF)

S7 Fig P2RX7-mediated IL-1β does not regulate *Ccl2/Ccl5* chemokine expression in HSCs. Relative expression of *CCL2* and *CCL5* in human primary HSCs cultured with CM from (A) CD14^+^ monocytes (CM-MO) and (B) Kupffer cells (CM-KC) treated with LPS, ATP ± SGM-1019 and/or IL-1Ra. (C) CCL2 and CCL5 levels in HSC cultured with conditioned media from CD14^+^ monocytes (CM-MO) and Kupffer cells (CM-KC) treated with LPS, ATP ± SGM-1019 and/or IL-1Ra. In all statistical plots, the data are shown as the mean ± SEM. n.s, for not significant. *P ≤ 0.05, **P ≤ 0.01, ***P ≤ 0.001, ****P ≤ 0.0001 by one-way ANOVA.
(TIFF)

S8 Fig SGM-1019 ameliorates hepatotoxicity in a chemically-induced liver fibrosis model in non-human primates. (A) Body weight of monkeys during the 6 weeks of treatments. (B) Liver weight of monkeys after 6 weeks of treatments. (C) Relative expression of *TGFβ1* (D) *CCL2* and *CCL5* in the livers from the six groups of monkeys. In all statistical plots, the data are shown as the mean ± SEM. *P ≤ 0.05, **P ≤ 0.01, ***P ≤ 0.001, ****P ≤ 0.0001 by one-way ANOVA.
(TIFF)

S1 File (PDF)
