# Supplementary material for: Pharmacological inhibition of P2RX7 ameliorates liver injury by reducing inflammation and fibrosis
Source: PLoS One. 2020 Jun 3;15(6):e0234038. doi: 10.1371/journal.pone.0234038 (PMC7269334; doi:10.1371/journal.pone.0234038)
Supplement: S1 Table — (PDF) [file pone.0234038.s001.pdf]

| Donor code | Category | Steatosis grade | Inflammation score | Fibrosis score | NAFLD activity score |
|---|---|---|---|---|---|
| **H80** | **Healthy** | **0/3** | **0/4** | **0/4** | **0/8** |
| **H57** | **Healthy** | **0/3** | **0/4** | **0/4** | **0/8** |
| **H63** | **Healthy** | **0/3** | **0/4** | **0/4** | **0/8** |
| **H26** | **Healthy** | **0/3** | **0/4** | **0/4** | **0/8** |
| **H33** | **Healthy** | **0/3** | **0/4** | **0/4** | **0/8** |
| **H70** | **Healthy** | **0/3** | **0/4** | **0/4** | **0/8** |
| **H64** | **NASH** | **2/3** | **2/4** | **1/4** | **5/8** |
| **H76** | **NASH** | **3/3** | **1/3** | **2-3/4** | **5/8** |
| **H59** | **NASH** | **3/3** | **0-1/4** | **1/4** | **4/8** |
| **H21** | **NASH** | **3/3** | **1/4** | **1a/4** | **5/8** |
| **H76** | **NASH** | **3/3** | **1/3** | **2-3/4** | **5/8** |
| **H22** | **NASH** | **3/3** | **1/4** | **2/4** | **5/8** |
| **H66** | **NASH** | **3/3** | **2/4** | **2/4** | **6/8** |
