# Supplementary figures and images for: Pharmacological inhibition of P2RX7 ameliorates liver injury by reducing inflammation and fibrosis
Source: PLoS One. 2020 Jun 3;15(6):e0234038. doi: 10.1371/journal.pone.0234038 (PMC7269334; doi:10.1371/journal.pone.0234038)

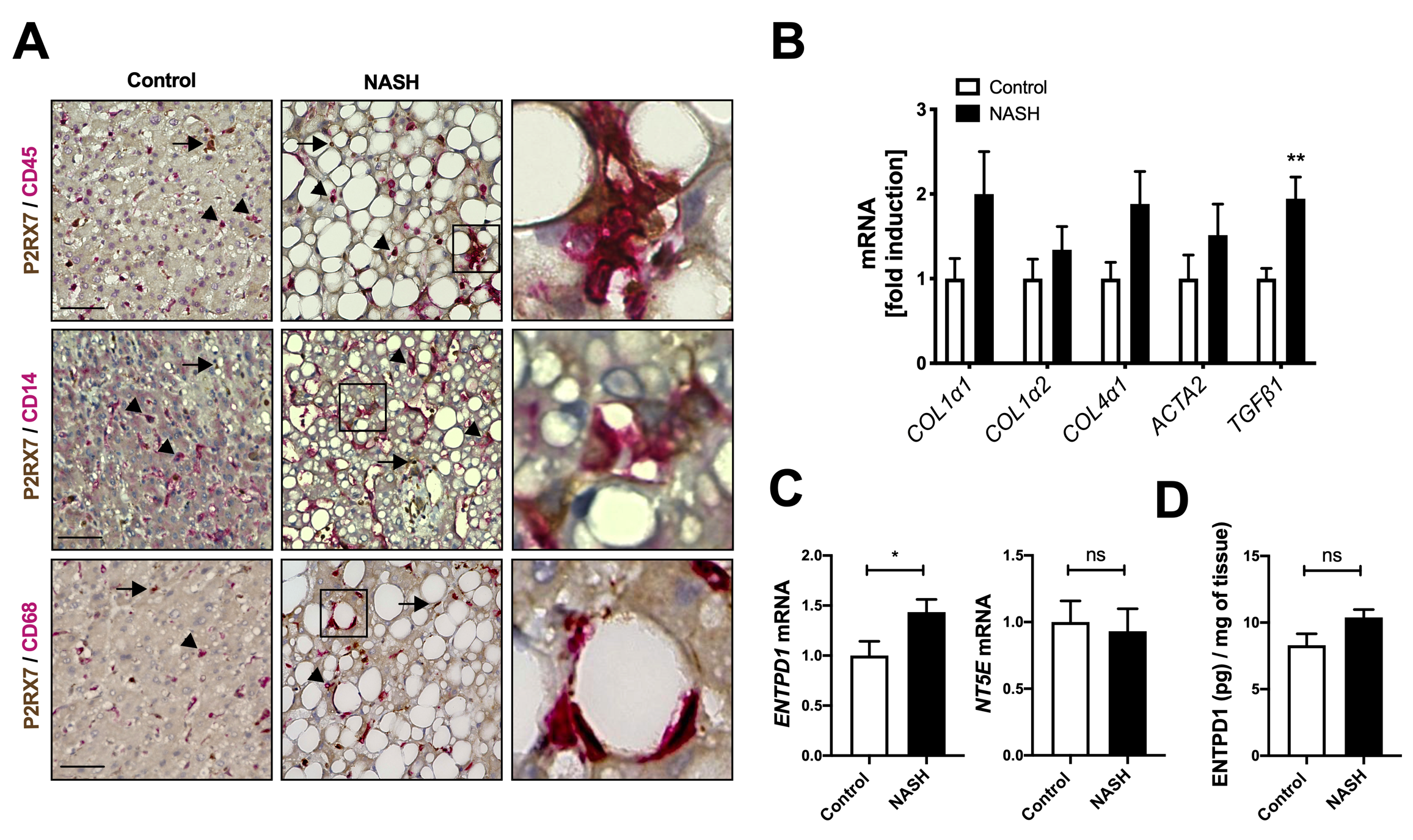

Supplement: S1 Fig — (A) Representative images (objective 40X) of immunohistochemical staining of P2RX7 (brown) with CD45, CD14, or CD68 (red) in liver tissue from a representative control and NASH donor. Scale bar, 100 μM. Black arrows highlight P2RX7+ cells and black arrowheads highlight CD45+, CD14+, and CD68+ cells. Area in square is shown amplified on right column images. (B) Relative expression levels of COL1α1, COL1α2, COL4α1, ACTA2 and TGFβ in liver tissue from control and NASH donors (n = 5 individuals per group). (C) Relative expression levels of ENTPD1 and NT5E and (D) ENTPD1 levels in liver tissue from control and NASH donors (n = 5 individuals per group). In all statistical plots, the data are shown as the mean ± SEM. *P ≤ 0.05, **P ≤ 0.01, ***P ≤ 0.001, ****P ≤ 0.0001 by two-sided Student’s t-test. (TIFF) [file pone.0234038.s002.tiff]

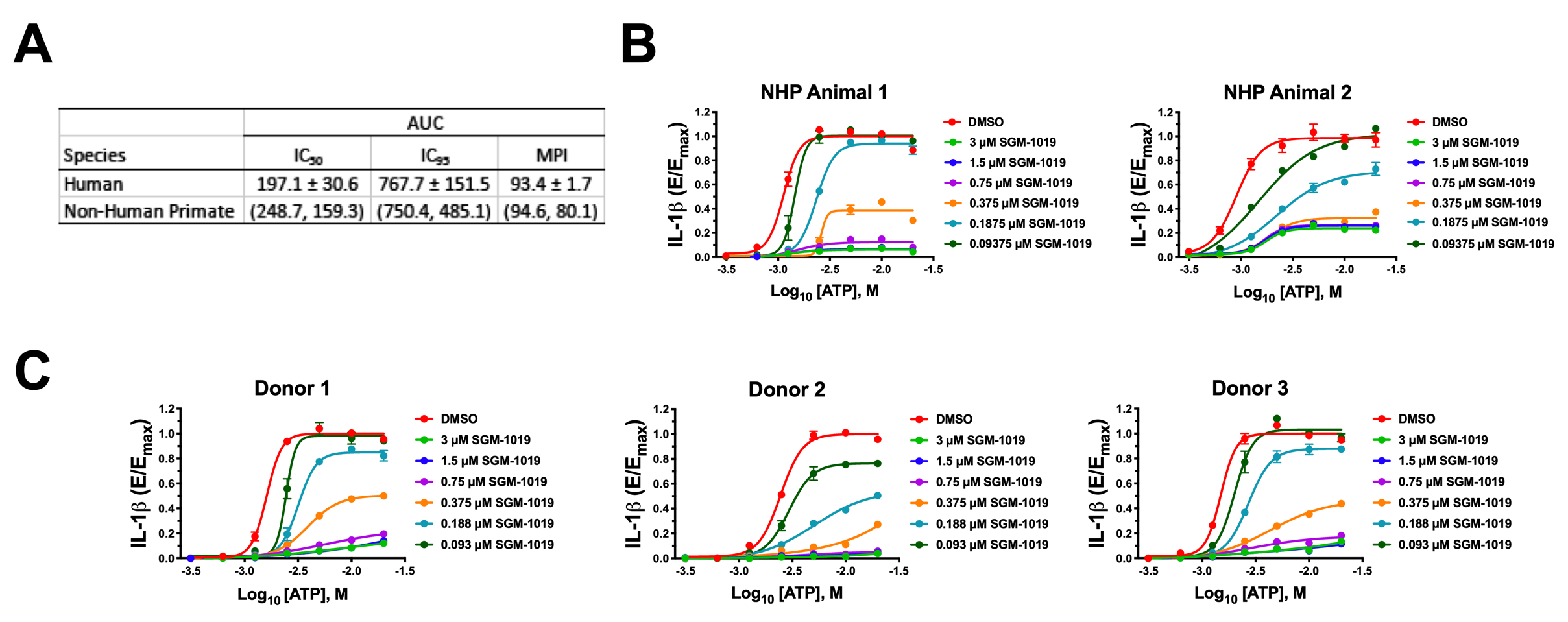

Supplement: S2 Fig — (A) Species comparison of SGM-1019 in an ex vivo whole blood ATP-dependent IL-1β release assay. Table shows inhibitory concentration (IC) of SGM-1019 at 50% (IC50), 95% (IC95), and maximum percent inhibition (MPI) of SGM-1019 in human and primate blood. AUC = Area under curve. Data in nanomolar (nM) is shown as the mean ± SEM (human) and individual replicates (NHP). Effect of SGM-1019 (0–3 μM) on LPS/ATP induced IL-1β secretion expressed as a proportion of IL-1β secretion of vehicle treated LPS/ATP primed blood (E/Emax) in (B) non-human primate (n = 2 monkeys) and (C) human (n = 3 individuals). Blood was treated with LPS plus SGM-1019 for 1 hour (human) or 2 hours (NHP) prior to the addition of ATP (0–20 mM) for 45 min. (TIFF) [file pone.0234038.s003.tiff]

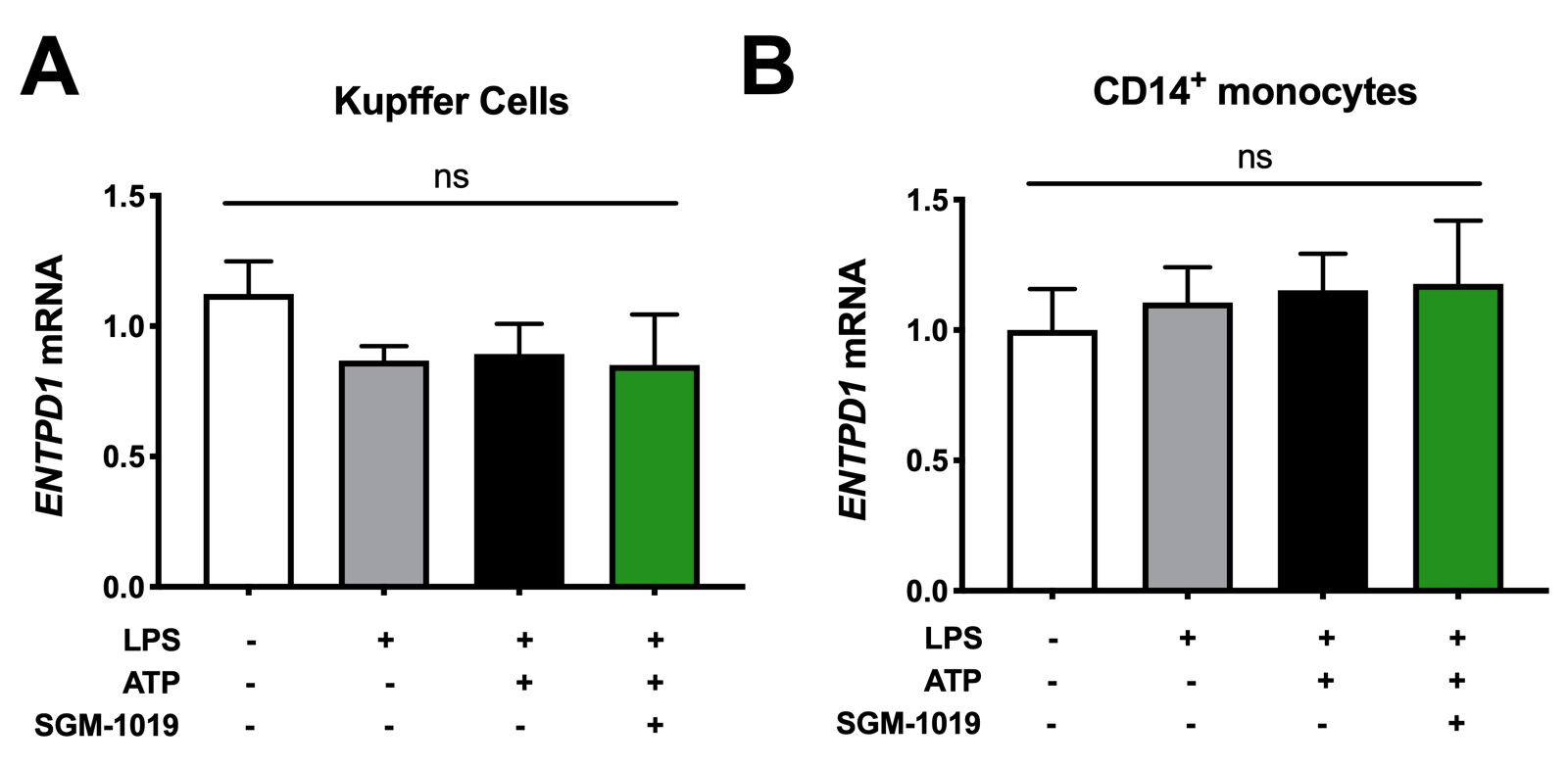

Supplement: S3 Fig — Relative expression of ENTPD1 in (A) KC and (B) CD14+ monocytes treated with LPS, ATP ± SGM-1019. In all statistical plots, the data are shown as the mean ± SEM. n.s, for not significant. *P ≤ 0.05, **P ≤ 0.01, ***P ≤ 0.001, ****P ≤ 0.0001 by one-way ANOVA. (TIFF) [file pone.0234038.s004.tiff]

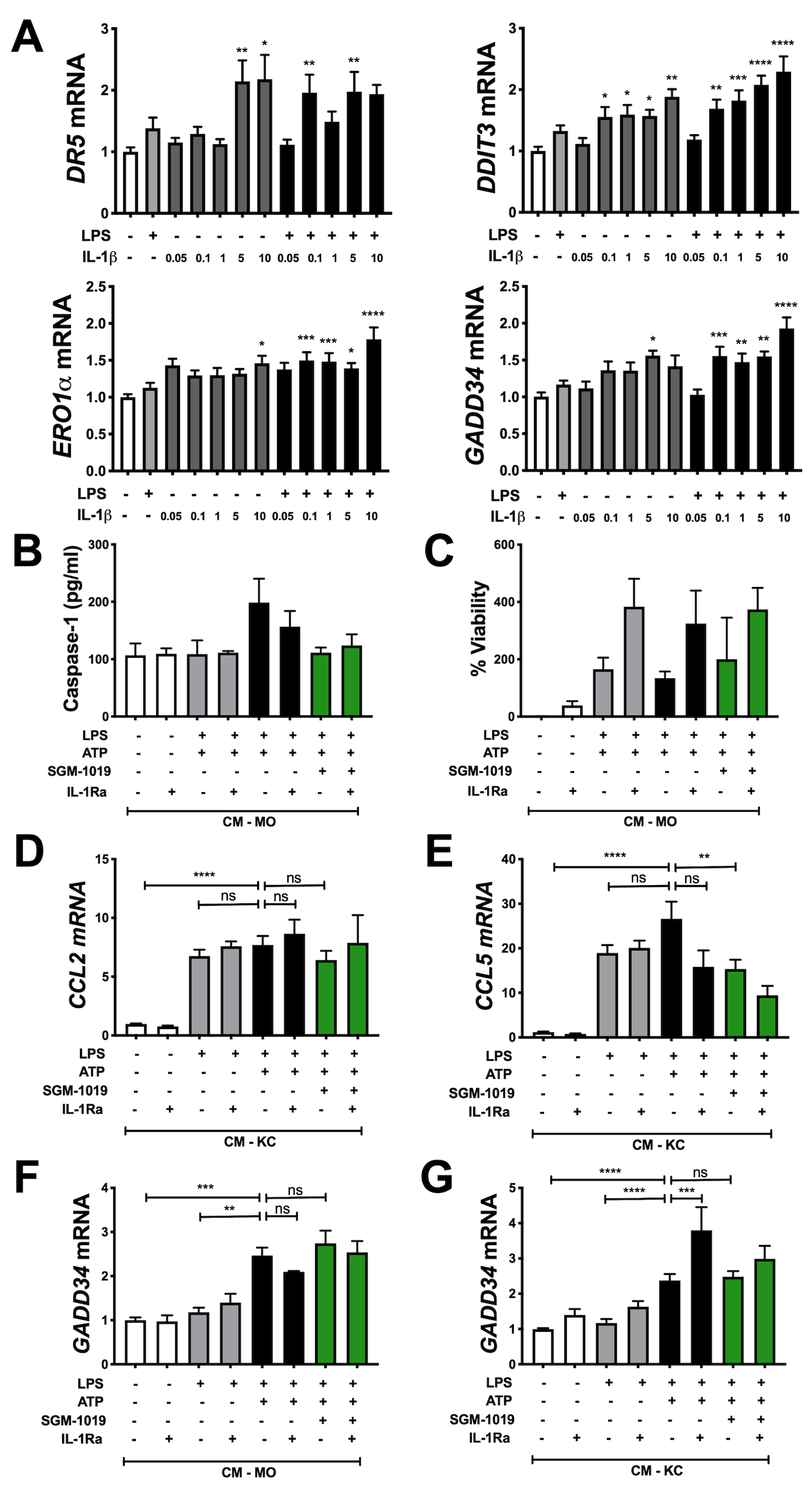

Supplement: S4 Fig — (A) Relative expression of ER stress genes DR5, DDIT3, ERO1α, and GADD34 in human primary hepatocytes treated with LPS (100 ng/ml) with or without increasing concentrations of IL-1β. (B) Caspase-1 and (C) Percentage of viability of human primary hepatocytes cultured with CM from CD14+ monocytes (CM-MO) treated with LPS, ATP ± SGM-1019 and/or IL-1Ra. Relative expression of (D) CCL2 and (E) CCL5 in hepatocytes cultured with CM from Kupffer cells (CM-KC) treated with LPS, ATP ± SGM-1019 and/or IL-1Ra. Relative expression of GADD34 in human primary hepatocytes cultured with CM from (F) CD14+ monocytes (CM-MO) and (G) Kupffer cells (CM-KC) treated with LPS, ATP ± SGM-1019 and/or IL-1Ra. In all statistical plots, the data are shown as the mean ± SEM. n.s, for not significant. *P ≤ 0.05, **P ≤ 0.01, ***P ≤ 0.001, ****P ≤ 0.0001 by one-way ANOVA. (TIFF) [file pone.0234038.s005.tiff]

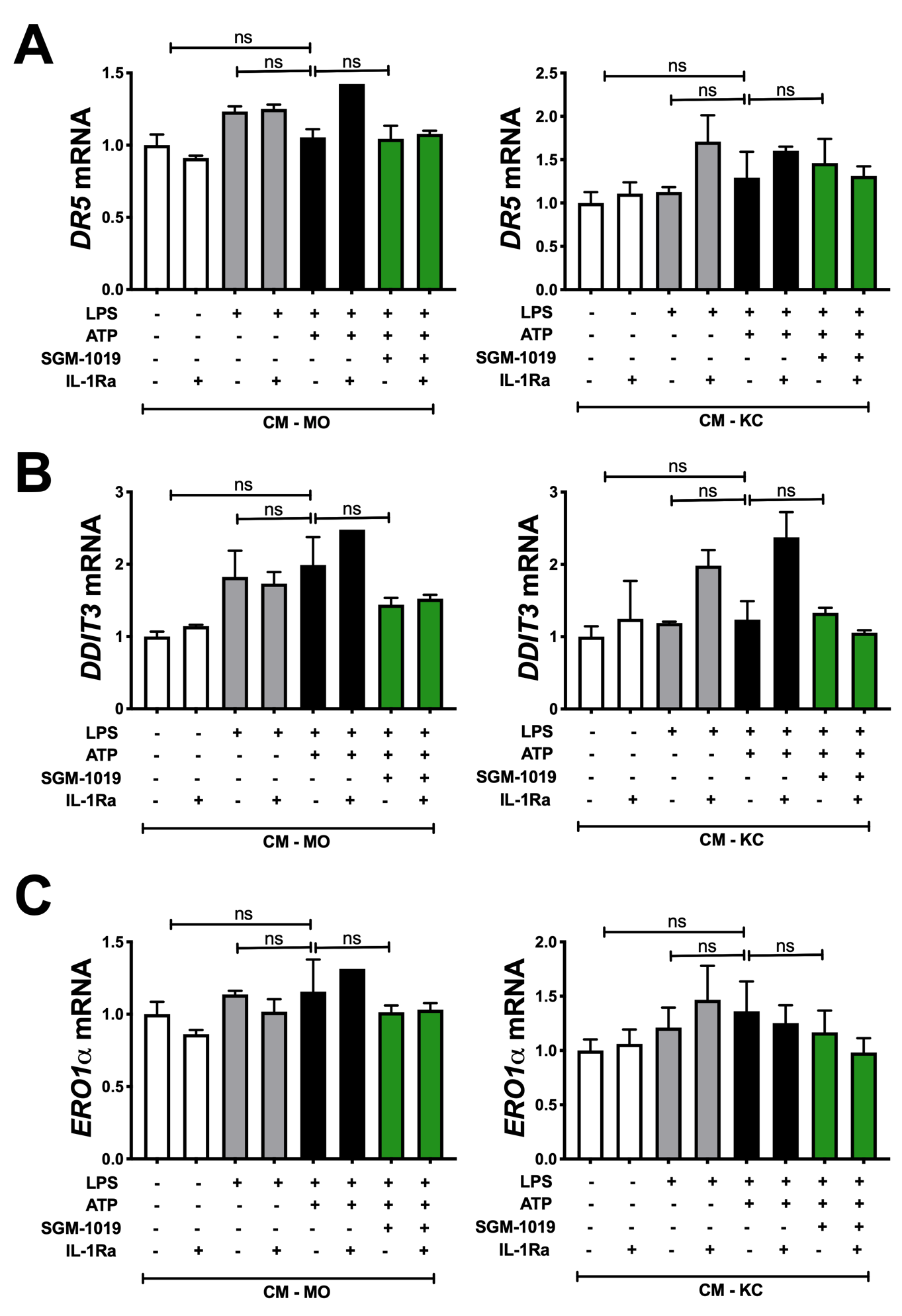

Supplement: S5 Fig — Relative expression of ER stress genes (A) DR5, (B) DDIT3, and (C) ERO1α in human primary hepatocytes cultured with CM from CD14+ monocytes (CM-MO) or with CM from Kupffer cells (CM-KC) both treated with LPS, ATP ± SGM-1019 and/or IL-1Ra. In all statistical plots, the data are shown as the mean ± SEM. n.s, for not significant. *P ≤ 0.05, **P ≤ 0.01, ***P ≤ 0.001, ****P ≤ 0.0001 by one-way ANOVA. (TIFF) [file pone.0234038.s006.tiff]

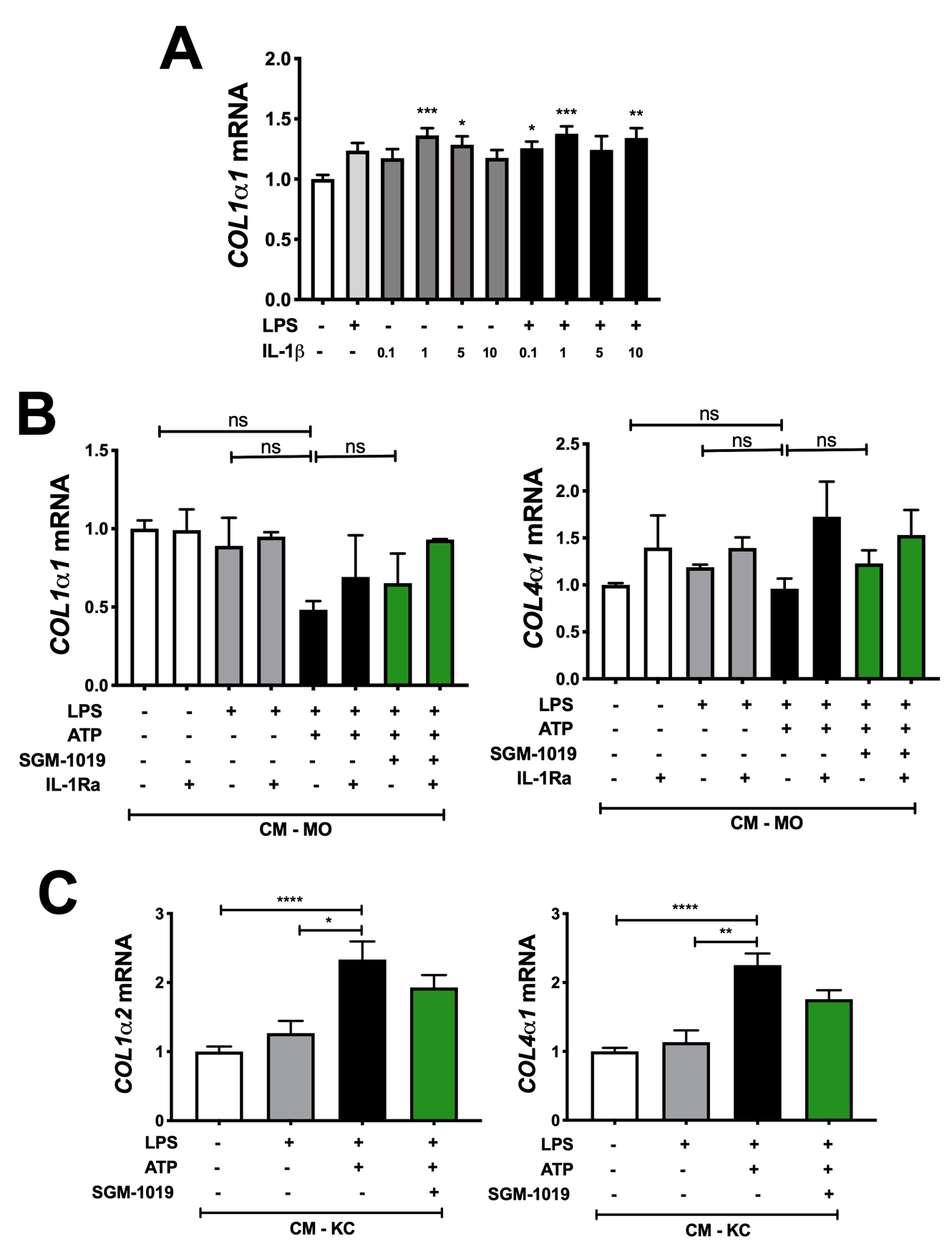

Supplement: S6 Fig — (A) Relative expression of COL1α1 in HSC treated with LPS ± increasing concentrations of rhIL-1β (IL-1β). (B) Relative expression of COL1α1 and COL4α1 in HSC cultured with conditioned media from CD14+ monocytes treated with LPS, ATP ± SGM-1019 (CM-KC). (C) Relative expression of COL1α2 and COL4α1 in HSC cultured with conditioned media from Kupffer cells treated with LPS, ATP ± SGM-1019 (CM-KC). In all statistical plots, the data are shown as the mean ± SEM. n.s, for not significant. *P ≤ 0.05, **P ≤ 0.01, ***P ≤ 0.001, ****P ≤ 0.0001 by one-way ANOVA. (TIFF) [file pone.0234038.s007.tiff]

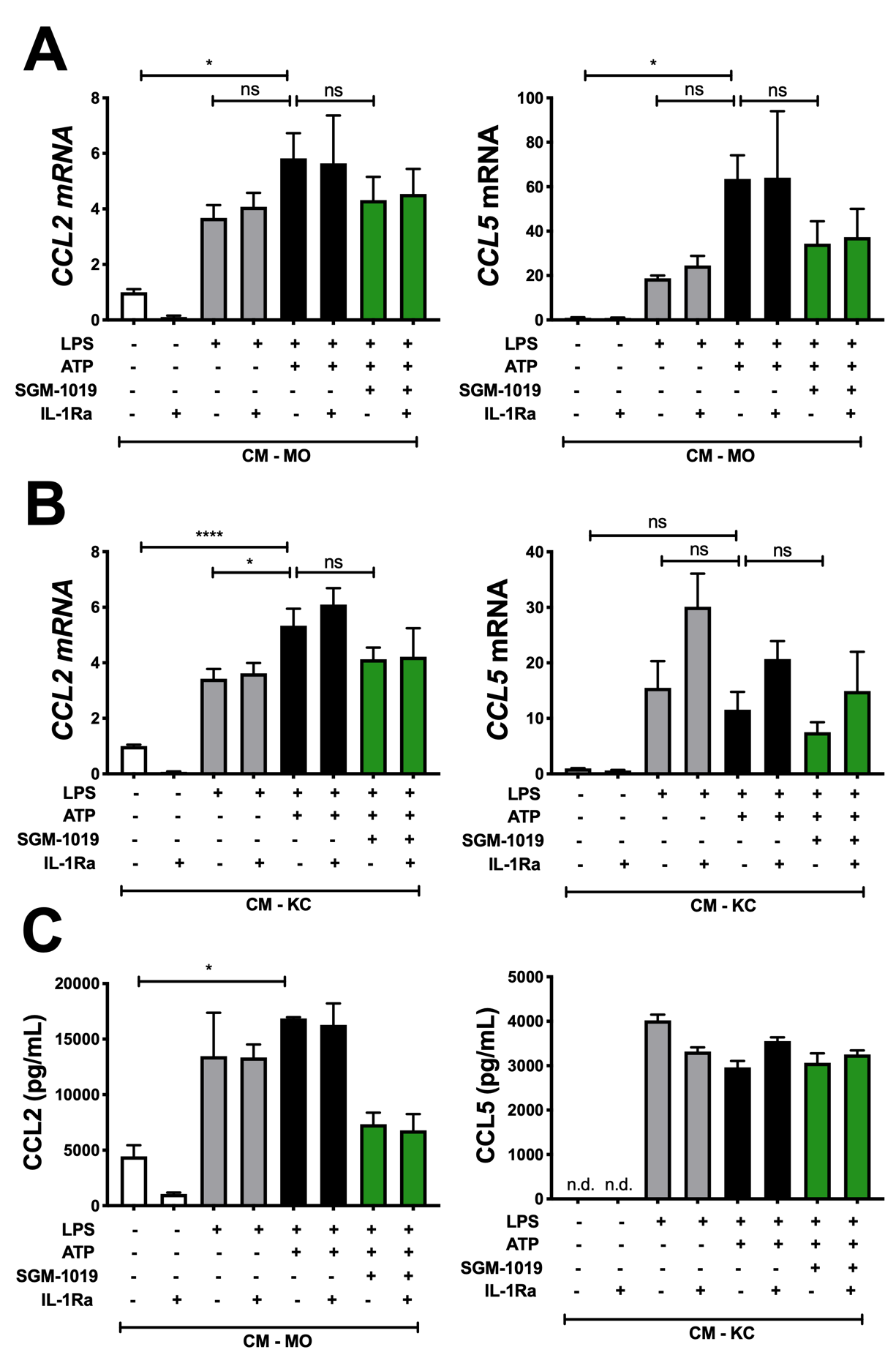

Supplement: S7 Fig — Relative expression of CCL2 and CCL5 in human primary HSCs cultured with CM from (A) CD14+ monocytes (CM-MO) and (B) Kupffer cells (CM-KC) treated with LPS, ATP ± SGM-1019 and/or IL-1Ra. (C) CCL2 and CCL5 levels in HSC cultured with conditioned media from CD14+ monocytes (CM-MO) and Kupffer cells (CM-KC) treated with LPS, ATP ± SGM-1019 and/or IL-1Ra. In all statistical plots, the data are shown as the mean ± SEM. n.s, for not significant. *P ≤ 0.05, **P ≤ 0.01, ***P ≤ 0.001, ****P ≤ 0.0001 by one-way ANOVA. (TIFF) [file pone.0234038.s008.tiff]

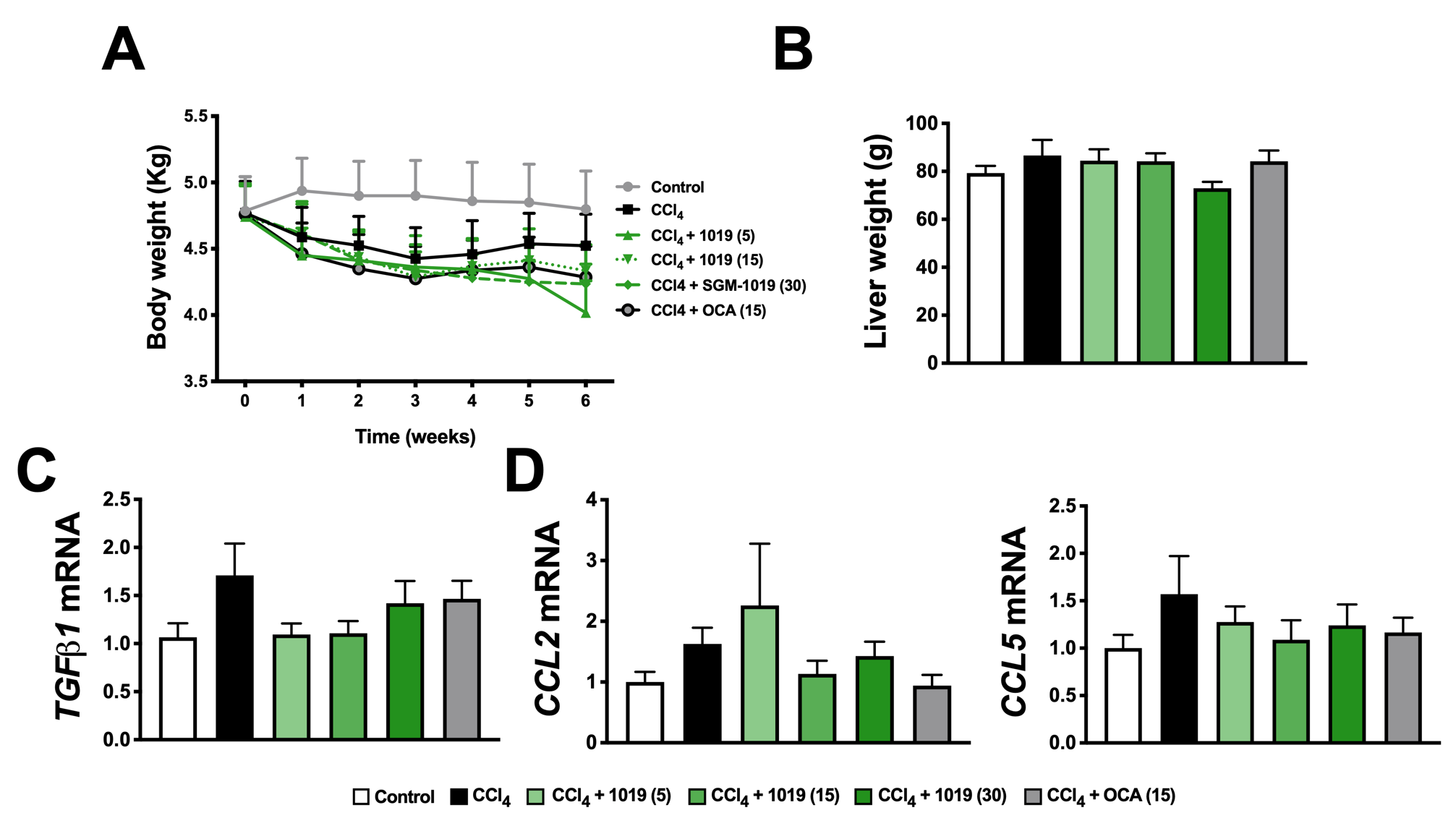

Supplement: S8 Fig — (A) Body weight of monkeys during the 6 weeks of treatments. (B) Liver weight of monkeys after 6 weeks of treatments. (C) Relative expression of TGFβ1 (D) CCL2 and CCL5 in the livers from the six groups of monkeys. In all statistical plots, the data are shown as the mean ± SEM. *P ≤ 0.05, **P ≤ 0.01, ***P ≤ 0.001, ****P ≤ 0.0001 by one-way ANOVA. (TIFF) [file pone.0234038.s009.tiff]
