# Supplementary material for: Pharmacological inhibition of P2RX7 ameliorates liver injury by reducing inflammation and fibrosis
Source: PLoS One. 2020 Jun 3;15(6):e0234038. doi: 10.1371/journal.pone.0234038 (PMC7269334; doi:10.1371/journal.pone.0234038)
Supplement: S1 File — (PDF) [file pone.0234038.s010.pdf]

Supplementary Materials and Methods

Non-human primate study

All animals need to be euthanized as long as any of the following conditions are met.

a. Weight loss: Loss of 20% of the original body weight, cachexia, or consumptive symptoms. The weight loss of non-growth animals can be based on the weight or average body weight of the monkeys just entering the facility; the body weight of the monkeys in the growing season may not decrease, but if it is unable to gain weight, it is still considered to be weight loss.
b. Loss of appetite: monkeys do not eat at all for 5 days; lose appetite for 7 days (uptake is less than 50% of normal).
c. Weak/dying state: monkeys which are obviously weak without anesthesia or sedation, they can barely stand after they stand up, or they have almost no response to external stimuli, and their body temperature drops.
d. Infection: caused by an increase in the number of white blood cells, an increase in body temperature, and accompanied by systemic symptoms.
e. When the clinical symptoms of severe organ loss have occurred and the treatment is ineffective or the veterinarian judges that the prognosis is poor:

f. Respiratory system: difficulty breathing, cyanosis.
g. Cardiovascular system: Large blood loss, anemia still given after an in- fusion treatment.

and appropriate sedation, analgesia or anesthesia (ketamine 10 mg/kg, IM) will be applied.

If a consensus cannot be reached in determining if an animal needs to be removed from a study or euthanized, or in the absence of an investigator, the oversight bodies – Institutional official, attending veterinarian and IACUC would convene a meeting with concerned groups to arrive at a final course of action. The ultimate authority and responsibility are given to the attending veterinarian.
